# Early-Life Environmental and Child Factors Associated with the Presence of Disruptive Behaviors in Seven-Year-Old Children with Autistic Traits in the Avon Longitudinal Study of Parents and Children

**DOI:** 10.1007/s10803-021-05081-x

**Published:** 2021-07-10

**Authors:** Simone Breider, Pieter J. Hoekstra, Klaas J. Wardenaar, Barbara J. van den Hoofdakker, Andrea Dietrich, Annelies de Bildt

**Affiliations:** 1 Department of Child and Adolescent Psychiatry, University Medical Center Groningen, University of Groningen, Lübeckweg 2, 9723 HE Groningen, The Netherlands; 2 Department of Psychiatry, University Medical Center Groningen, University of Groningen, Groningen, The Netherlands; 3 Department of Clinical Psychology and Experimental Psychopathology, University of Groningen, Groningen, The Netherlands

**Keywords:** ALSPAC, Autism spectrum disorder, Disruptive behaviors, Risk factors, Early-life, Environmental

## Abstract

We studied the association of early-life environmental and child factors with disruptive behaviors in children with autistic traits around age 7, in the Avon Longitudinal Study of Parents and Children (*n* = 6,401). Logistic regression with the least absolute shrinkage and selection operator indicated that disruptive behaviors were associated with prenatal smoking, no seafood-consumption during pregnancy, breech presentation at delivery, neonatal feeding problems, low social-economic situation, suboptimal preschool family environment, maternal depression, maternal antisocial behavior, male sex, and difficult child temperament. Compared to controls, male sex, maternal depression, and suboptimal preschool family environment were related to autistic traits without disruptive behaviors. Thus, there may be a difference in early-life factors related to autism spectrum disorder with and without disruptive behaviors.

**Supplementary Information:**

The online version contains supplementary material available at 10.1007/s10803-021-05081-x.

Many children with autism spectrum disorder (ASD) frequently display disruptive behaviors, including anger, disobedience, and refusal to comply with rules: prevalence estimates vary across behaviors and studies, with estimates of approximately 50% for temper tantrums, 30% for defiant behavior, and 20% for aggressive behavior (Chandler et al., 2016; Kantzer et al., 2018; Lecavalier, 2006; Maskey et al., 2013; Simonoff et al., 2008). Disruptive behaviors have repeatedly been related to unfavorable outcomes in children with ASD, e.g., social skills, to parental stress, and to decreased family functioning (Huang et al., 2014; Matson et al., 2010; Sikora et al., 2013; Tomanik et al., 2004). A vast body of research points to the importance of early-life environmental and child factors for ASD (Baio et al., 2018; Charman & Chakrabarti, 2016; Hallmayer et al., 2011; Mandy & Lai, 2016; Vijayakumar & Judy, 2016) and disruptive behaviors in children without ASD (Carneiro et al., 2016; Guney et al., 2015; Latimer et al., 2012). However, studies on early-life and child factors related to disruptive behaviors in children with ASD are scarce.

The few available studies on early-life environmental factors associated with the presence of disruptive behaviors in ASD indicated involvement of pregnancy complications (Gadow et al., 2008a) and parental variables such as mental health problems, parental stress, and less warm and more negative/harsh parenting (Bauminger et al., 2010; Gadow et al., 2008a; Maljaars et al., 2014; McRae et al., 2019; Midouhas et al., 2013; Visser et al., 2013). Furthermore, family demographic factors associated with disruptive behaviors in offspring with ASD may include single parenthood, low social economic status, large family size, and adolescent pregnancy (Chandler et al., 2016; Gadow et al., 2008a; Midouhas et al., 2013; Simonoff et al., 2008; St. Pourcain, 2011). Finally, child factors that have been related to disruptive behaviors in ASD include white ethnicity (Gadow et al., 2008a) and intelligence (although inconsistent in its direction across studies; Cervantes et al., 2014; Chandler et al., 2016; Presmanes Hill et al., 2014). However, existing research on disruptive behaviors in ASD often used small samples, was based on retrospective measurements, or investigated a limited set of factors.

In studies on early-life factors associated with the presence of ASD, disruptive behaviors are not consistently reported on or controlled for. Thus, some of the factors previously found associated with ASD may specifically be related to ASD *with* disruptive behaviors, and others to ASD *without* disruptive behaviors. Furthermore, it may be that factors associated with the presence of disruptive behaviors in children with ASD are also associated with the presence of ASD without disruptive behaviors. Currently, however, it is not well known how factors related to ASD with and without disruptive behaviors overlap.

A few studies have reported similarities in factors associated with the presence of disruptive behaviors in ASD and non-ASD populations (Gadow et al., 2008b; Mandy et al., 2014). However, for most factors that have been associated with disruptive behaviors in non-ASD samples, it is currently unclear whether they are associated with disruptive behaviors in children with ASD.

In the current study, we investigated a broad range of early-life environmental and child factors (i.e., from the prenatal period until age 4) in relation to the presence of disruptive behaviors in children with autistic traits at age 7. Specifically, we looked at family demographic, pregnancy, delivery, neonatal, child, and preschool (0–4 years) parental and family factors, in a large sample of children from the prospective Avon Longitudinal Study of Parents and Children (ALSPAC; Boyd et al., 2013; Fraser et al., 2013). In order to investigate factors associated with the presence of disruptive behaviors in children with autistic traits, we compared children with autistic traits with and without co-occurring disruptive behaviors. We also made comparisons with unaffected controls, i.e., children without autistic traits and no disruptive behaviors, to investigate the overlap in factors associated with autistic traits *with* and *without* disruptive behaviors. We selected the variables in our model based on factors previously associated with disruptive behaviors in both ASD and non-ASD populations and factors previously associated with ASD, aiming to provide a synthesis of potential early-life factors associated with the presence of disruptive behaviors in ASD.

## Methods

### Participants

Participants were 6,401 children from the ALSPAC study, a birth cohort based on pregnant women with expected delivery dates from April 1991 to December 1992 from the county of Avon, UK (Boyd et al., 2013; Fraser et al., 2013). Information on participants’ health and development has been collected since pregnancy and data collection is still ongoing. ALSPAC initially recruited 14,541 pregnant women and has bolstered the sample when the oldest children were approximately 7 years of age. The total sample size for analyses using any data collected after the age of seven is therefore 15,454 pregnancies, resulting in 15,589 fetuses. Of these 14,901 were alive at 1 year of age.

We included three groups: children around age 7 with (i) autistic traits and disruptive behaviors (ASD + DB; *n* = 307), (ii) autistic traits without disruptive behaviors (ASD − DB; *n* = 198), and (iii) no autistic traits and no disruptive behaviors (controls; *n* = 5,896). Presence of autistic traits was based on the Social Communication Disorder Checklist (Skuse et al., 1997) and presence of disruptive behaviors was based on the Strengths and Difficulties Questionnaire (Goodman, 1997). From the total ALSPAC sample, we excluded children for whom no data were available on autistic traits or disruptive behavior (53.2%), who had an IQ below 70 (0.9%; to prevent mislabeling of social and communication difficulties due to low cognitive capabilities as autistic traits), or who were the second born part of a twin (1.3%; to ensure independent observations).

### Measurements

#### Dependent Variable

Our dependent variable was defined as group membership of one of the three groups: ASD + DB_,_ ASD − DB, and controls. Autistic traits were measured with the Social Communication Disorder Checklist (SCDC; Skuse et al., 1997), in line with earlier ALSPAC studies into ASD (e.g., St. Pourcain et al., 2011; Stergiakouli et al., 2017), and assessed at the child’s age of 7 years and 7 months. Mothers rated 12 items, e.g., ‘Not aware of other peoples’ feelings’, ‘Does not seem to understand social skills’, and ‘Cannot follow a command unless it is carefully worded’ on a 3-point scale (0 = ‘not true’, 1 = ‘quite or sometimes true’, 2 = ‘very or often true’). Sufficient internal consistency (Cronbach’s α = 0.93; current study: α = 0.89, *n* = 6,401) and test–retest reliability (intraclass correlation = 0.81) have been reported (Skuse et al., 2005). Children with an SCDC score ≥ 9 were assigned to an autistic traits group, ‘ASD group’ for conciseness. This cut-off has been shown useful to discriminate children with a diagnosis of ASD from children from the general population and from children with other clinical diagnoses (e.g., conduct disorder and attention-deficit hyperactivity disorder; sensitivity = 0.90; specificity = 0.69; Skuse et al., 2005).

The level of disruptive behaviors was assessed with the externalizing subscale of the Strengths and Difficulties Questionnaire (SDQ_EXT_; Goodman, 1997; Goodman et al., 2010), i.e., the sum of the Hyperactivity and the Conduct problems scale, at the child’s age of 6 years and 9 months. Mothers rated the items (five per scale) on a 3-point scale (0 = ‘not true’, 1 = ‘somewhat true’, 2 = ‘certainly true’). The externalizing subscale has been suggested as a justified and valid measure of externalizing problems in the general population (Goodman et al., 2010). Internal consistency (Cronbach’s α = 0.63 for conduct problems, α = 0.77 for hyperactivity; current study: α SDQ_EXT_ = 0.72, *n* = 6,401) and test–retest stability (0.64 for conduct problems and 0.72 for hyperactivity) have been reported as generally satisfactory in a community sample (Goodman, 2001). A score of ≥ 3 on the SDQ Conduct problems scale and of ≥ 6 on the SDQ Hyperactivity scale have been proposed to distinguish between ‘normal’ and ‘borderline/abnormal’ (i.e., the highest 20%) scores in the community (Goodman, 1997). SDQ_EXT_ ≥ 9 (i.e., 3 + 6) was therefore used to differentiate children with and without disruptive behaviors.

#### Independent Variables

Table 1 shows the independent variables that we included in the statistical models, divided over six categories: (1) family demographics, (2) pregnancy variables, (3) delivery variables, (4) neonatal variables, (5) child variables, and (6) preschool parental and family variables (measured before the age of 4 years). For most variables, information was collected prospectively with questionnaires completed by the participant’s mother. Other sources of information included partner-rated questions and clinical records of the mother. Continuous variables were coded such that a higher score signifies a higher level (e.g., a higher score on maternal anxiety signifies more anxiety; a higher score on intelligence signifies a higher IQ). Regarding preschool parental and family variables, parenting score indicates the frequency with which parents do activities with their child (e.g., playing, reading, and walking). Parental bonding signifies the degree of parental enjoyment and confidence regarding parenting and the child. The way parents experience being a parent was measured with the positive and negative parenting experience scores and the quality of the relationship that parents have with their child was measured with the positivity and negativity scale scores.

**Table 1:** Family demographic, pregnancy, delivery, neonatal, child, and preschool parental and family factors

Family demographics	Maternal age at time of birth (Low [< 20], Middle [20–29], or High [> 29]), High partner’s age at time of birth (≥ 35), Firstborn child, Summer birth, Twin birth, Maternal and Partner’s education (CSE/None, Vocational, O level, A level, or Degree; measured during pregnancy), Maternal and Partner’s social class (High, Medium, or Low; measured during pregnancy), Single household at 8, 21, 33, and 47 months, and Number of child’s siblings at 18 and at 30 months^c^
Pregnancy	Infections in first trimester, Infections in second trimester, Infections in third trimester, Maternal alcohol use, Smoking mother, Smoking partner, Maternal medication use, Maternal antidepressant use, Diabetes, Maternal pre-pregnancy weight^c^, High level of street traffic, Weekly seafood consumption, Folic acid intake, Maternal prenatal anxiety^c^, Maternal prenatal depression^c^, Prenatal stress (life events)^c^, Maternal external locus of control^c^, Affection between parents^c^, Aggression between parents^c^, Maternal non-positive pregnancy feelings, Pre-eclampsia, Vaginal bleeding in pregnancy, Premature birth (< 37 weeks), and Low birthweight (< 2500 g)
Delivery	Breech presentation before labor*, Breech presentation at onset of labor*, Breech presentation at delivery/cesarean section*, Cesarean section*, Breech birth*, Maternal hemorrhage prior to delivery*, Precipitate labor*, and Umbilical cord complications*
Neonatal	Low Apgar at 5 min (< 7)*, Child’s age at discharge from hospital*^c^, Anemia, Feeding problems*, Oxygen problems*, Breastfeeding in first four weeks, and Duration of breastfeeding (never, < 3 months, 3–5 months, or ≥ 6 months)
Child	Intelligence^c^, Non-white ethnicity, Male sex, and Temperament scales 24 months^c^ (i.e., Activity, Rhythmicity (shows variability in behavior), Approach (difficulty with the unknown), Adaptability (difficulty with waiting or change), Intensity (reacts strongly), Mood (negative mood), Persistence (reduced attention span), Distractibility, and Threshold (quicker to notice things)
Preschool parental and family	Mother’s experienced social support (8 weeks–21 months)^c^, Affection between parents 8 months^c^ (mother and partner* rated), Aggression between parents 8 months^c^ (mother and partner* rated), Warmth between parents 33 months^c^ (mother and partner* rated), Rows between parents 33 months^c^ (mother and partner* rated), Maternal anxiety^c^, Partner’s anxiety^c^, Maternal depression^c^, Partner’s depression^c^ (anxiety and depression measured 8 weeks–21 months), Stress (life events; 21–33 months)^c^, Maternal lifetime antisocial behavior^c^, Accident prevention measures (in household; 8 months)^c^, Parenting score 6 months^c^ (mother and partner), Parenting score 18 months^c^ (mother and partner), Parenting score 38 months^c^ (mother and partner), Parental bonding 8 months^c^ (mother and partner*), Parental bonding 33 months mother^c^, Positive parenting experience score 21 months^c^ (mother and partner*), Negative parenting experience score 21 months^c^ (mother and partner*), Positivity scale score 47 months^c^ (mother and partner*), Negativity scale score 47 months^c^ (mother and partner*), and Child maltreatment (8–47 months)

*Variable with > 30% missing values

^c^Continuous variable

Parental education: *CSE* certificate of secondary education or general certificate of secondary education (GCSE) D–G *O level* Ordinary level or GCSE A–C *A level* Advanced level, *Degree* University degree

Online Resource Table 1 provides information per variable on data collection (measurement method, time-point, and informant) and on how derived variables were computed for the current study. Please note that the ALSPAC website contains details of all data that is available through a fully searchable data dictionary and variable search tool (http://www.bristol.ac.uk/alspac/researchers/our-data/).

### Statistical Analyses

#### Missing Values

If 50% or more of item-responses were missing, the SCDC score and SDQ scale scores were not calculated and the participant was not included in the study sample. If a participant missed less than 50%, missing item-responses were replaced by the participants mean score of the other items (i.e., use of ALSPAC’s ‘prorated’ scores; for the SCDC in 2.4% of participants, for the SDQ in 5.3% of participants).

Across all 100 independent variables, the total percentage of missing values was 11.7%, i.e., from the 640,100 cells (*n* = 6,401 × 100 variables) 11.7% of the cells were empty. Only 569 participants had no missing values. Since our intended method of analysis (see below) removes cases with missing data, missing values on independent variables had to be imputed. To limit bias in the imputations, we brought down the total percentage of missing values from 11.7 to 5% in two different ways, assuming that 5% of missing values would lead to a more reliable imputation than 11.7%. First, we reduced missing values by selecting a subset of participants with fewer than 13 missing values on the 100 independent variables, leaving a subset of 3,683 participants (i.e., subset A; *n* ASD + DB = 178, *n* ASD − DB = 120, and *n* controls = 3,385). Second, from the dataset of 6,401 participants and 100 independent variables, we removed independent variables with 30% or more missing values, resulting in a dataset with 79 independent variables (removed variables are marked with an * in Table 1; i.e., subset B). That is, the choice of selecting participants with fewer than 13 missing values (subset A) and variables with less than 30% missing values (subset B) was informed by how many participants/variables had to be removed to achieve a total percentage of missing values (i.e., cells) of 5%. Assuming that data were missing at random, both subsets were imputed ten times using multiple imputation in IBM SPSS Statistics (v25). We did not impute independent or dependent values of ALSPAC participants without a (prorated) SCDC and SDQ_EXT_ score, since generally, these participants missed data on many independent variables, which would make imputation unreliable.

#### Selection Bias

To explore selection bias, we compared participants in subset A (*n* = 3,683) to participants who were excluded from that subset due to high levels of missing data on independent variables (*n* = 6,401–3,683 = 2,718) on demographics (across the three study groups), and autistic traits and disruptive behavior (per study group). Furthermore, we compared the demographics of our total study sample (i.e., *n* = 6,401) to those of the subjects in the ALSPAC database that could not be allocated to one of our three groups due to missing data on the SCDC and/or SDQ around age 7 (*n* = 8,031). Missing data on the SCDC and/or SDQ was mainly due to increasing attrition in the ALSPAC study over time. Approximately 8,250 ALSPAC participants completed the questionnaire that included the SCDC at 7 years and 7 months, and approximately 8,500 completed the questionnaire that included the SDQ at 6 years and 9 months; see Boyd et al., 2013 for an overview of attrition in the ALSPAC study. To explore selection bias, *T*-tests and Chi-square tests were applied to the non-imputed data.

#### Lasso Analyses

Logistic regression with the least absolute shrinkage and selection operator (lasso; Tibshirani, 1996) was run in R (R Core Team, 2017) using the ‘glmnet’ R-package (Friedman et al., 2017) to identify the independent variables that show the largest associations with the dependent variable (i.e., group membership). Lasso is a shrinkage technique that enables estimation of regularized regression model solutions, in which only the strongest effects remain (i.e., are biased upwards) and the coefficients of weaker variables are shrunk to zero by penalty (Tibshirani, 1996). The size of the penalty is selected using a tuning parameter (lambda), the optimal value of which is determined through tenfold cross-validation. Since this process is subject to randomness, it was performed 1000 times, after which the lambda with the smallest mean error (difference between observed and estimated dependent variable values) was used to set the eventual model penalty. Given the binary scale of the used outcomes, a binomial link-function was used in all models.

Dependent variable categories were compared in pairs: (1) ASD + DB versus ASD − DB, (2) ASD + DB versus controls, and (3) ASD − DB versus controls. Comparison 1 was used to study factors related to the presence of disruptive behaviors in children with autistic traits. Through comparing the outcomes of comparison 1 and comparison 3, we examined whether factors related to disruptive behaviors in children with autistic traits (1) were also related to the presence of autistic traits without disruptive behaviors (3). Finally, through comparing the outcomes of comparison 2 and 3, we examined factors that were related to the presence of autistic traits with disruptive behaviors (2) and to the presence of autistic traits without disruptive behaviors (3), both in comparison to unaffected controls (i.e., children without autistic traits and no disruptive behaviors). We used unstandardized variables in all models, which enables a straightforward interpretation of individual variable results. For categorical independent variables, presence was coded as 1 and absence as 0. For categorical variables with more than two categories, dummy variables were created.

We explored linearity of the relationship between the independent variables and the log odds of the dependent variable using component residual plots in R (‘car’- package; Fox & Weisberg, 2011). Outliers were assessed through Cooks Distance.

Lasso analyses were conducted in the ten imputation sets of subset A (i.e., participants with a high amount of missing values excluded; *n* = 3,683; *n* ASD + DB = 178, *n* ASD − DB = 120, *n* controls = 3,385; 100 independent variables) and subset B (i.e., variables with a high amount of missing values excluded; *n* = 6,401; *n* ASD + DB = 307, *n* ASD − DB = 198, and *n* controls = 5,896; 79 independent variables). Because only the strongest effects remain in a lasso model and coefficients of other variables are shrunk to zero, our interest lay in whether a variable was kept in the model. Therefore, for each group comparison, an independent variable was assumed to be associated with group membership if it had a nonzero regression coefficient in six or more (i.e., more than half) of the ten imputation sets in *both* subsets, or, for variables that were only included in subset A, had a nonzero regression coefficient in six or more of the ten imputation sets of subset A. Lasso gives the percentage of explained deviation, i.e., the deviance of the formulated model relative to the deviance of an intercept-only model. The explained deviance can be seen as a measure of how good the fit of the model is (range 0–1; a higher score indicates better fit). To estimate the degree of association of early-life factors with autistic traits and disruptive behaviors, we calculated the minimum and maximum percentage of explained deviation across the ten imputation sets in both subsets, for each group comparison. Furthermore, we calculated the mean explained deviance of the six variable categories across the ten imputation sets in subset A, since this subset included all independent variables. To this end, we subtracted the explained deviance of all independent variables except those of the variable category of interest from the total explained deviance.

## Results

### Group Characteristics and Selection Bias

Table 2 shows the mean SCDC score (autistic traits) and SDQ_EXT_ score (disruptive behavior) around age 7, and, to describe the study sample, demographic characteristics per group in subset A (*n* ASD + DB = 178, *n* ASD − DB = 120, *n* controls = 3,385) and B (*n* ASD + DB = 307, *n* ASD − DB = 198, *n* controls = 5,896). As expected due to the use of cut-off scores, the mean SCDC score of participants in the unaffected controls group (i.e., children without autistic traits and no disruptive behaviors) was clearly below the mean score in both the ASD + DB group and the ASD – DB, and the mean SDQ_EXT_ score of the ASD + DB group was clearly above the mean of both the ASD − DB and the control group, in subset A as well as subset B. Additionally, the mean SCDC score was somewhat higher in the ASD + DB group than in the ASD − DB group, and the mean SDQ_EXT_ score was somewhat higher in the ASD − DB group than in the control group.

**Table 2:** Group characteristics in subsets A (participants with few missing values) and B (all participants), before imputation

Subset	ASD + DB *n* = 178 (A) *n* = 307 (B)		ASD – DB *n* = 120 (A) *n* = 198 (B)		Unaffected controls *n* = 3,385 (A) *n* = 5,896 (B)	
		*n*		*n*		*n*
SCDC: mean (SD)						
A^a^	13.0 (3.69)	178	11.3 (2.75)	120	1.80 (2.07)	3,385
B^b^	13.5 (4.10)	307	11.3 (2.82)	198	1.76 (2.07)	5,896
SDQ_EXT_: mean (SD)						
A^c^	11.8 (2.20)	178	6.00 (1.85)	120	3.83 (2.27)	3,385
B^d^	11.8 (2.25)	307	6.06 (1.93)	198	3.86 (2.28)	5,896
Sex: % male						
A	71.9	178	58.3	120	48.0	3,385
B	73.0	307	59.6	198	48.5	5,896
IQ: mean (SD)						
A	102 (17.8)	137	104 (17.3)	99	108 (15.5)	2,817
B	100 (17.2)	206	103 (17.0)	146	107 (15.3)	4,519
Social class mothers: % high						
A	39.4	155	41.2	102	43.7	2,997
B	33.9	245	38.1	160	41.1	4,793
Social class partners: % high						
A	38.6	158	50.0	102	47.5	3,117
B	37.2	253	50.6	160	44.8	5,062
Family: % two-parent household						
A	97.7	177	100	119	98.8	3,366
B	93.5	292	96.3	189	95.5	5,673
Ethnicity: % white						
A	96.6	178	98.3	119	97.5	3,364
B	96.6	295	97.4	194	96.5	5,710

High social class indicates a ‘professional’, ‘managerial’ or ‘technical’ occupation during pregnancy

Family: % two-parent household in the child’s first year

*ASD* Autism Spectrum Disorder, *DB* disruptive behavior, *SCDC* Social Communication Disorder Checklist (i.e., autistic traits), *SDQ*_EXT_ Sum of the Conduct and Hyperactivity subscale of the Strengths and Difficulties Questionnaire (i.e., disruptive behavior)

Except for the SCDC and SDQ_EXT_, group differences were not calculated, since demographic variables were included in the Lasso analyses

^a^ASD + DB/ASD − DB: *t* = − 4.40, *p* < 0.001; ASD + DB/Controls: *t* = − 66.8, *p* < 0.001; ASD − DB/Controls: *t* = − 48.6, *p* < 0.001

^b^ASD + DB/ASD − DB: *t* = − 6.64, *p* < 0.001; ASD + DB/Controls: *t* = − 90.7, *p* < 0.001; ASD − DB/Controls: *t* = − 63.0, *p* < 0.001

^c^ASD + DB/ASD − DB: *t* = − 23.5, *p* < 0.001; ASD + DB/Controls: *t* = − 45.4, *p* < 0.0001; ASD − DB/Controls: *t* = − 10.4, *p* < 0.001

^d^ASD + DB/ASD − DB: *t* = − 29.6, *p* < 0.001; ASD + DB/Controls: *t* = − 59.6, *p* < 0.001; ASD − DB/Controls: *t* = − 13.4, *p* < 0.001

With regard to selection bias, the mean SDQ_EXT_ and SCDC score in the three groups did not differ between participants who were included in subset A (*n* = 3,683) and those who were excluded from subset A due to a high amount of missing values on independent variables (*n* = 2,718; *n* ASD + DB = 129, *n* ASD − DB = 78, *n* controls = 2,511), with the exception of the mean SCDC score in the ASD + DB group (included 13.0, excluded 14.2; *t* = 2.53, *p* = 0.01; see Online Resource Table 2). Participants included in subset A did not differ on sex distribution compared to participants who were excluded from this subset. Yet, included participants scored higher on IQ, were more often of white ethnicity, from a higher social class, and from a two-parent household (see Online Resource Table 3). This was also the case when comparing our total study sample (*n* = 6,401, i.e., subset B) with ALSPAC subjects that were excluded due to missing data on the SCDC and/or SDQ (8,031 subjects; see Online Resource Table 4).

### Lasso Analyses

Component residual plots indicated that the assumption of linearity of the relationship between the independent variables and outcomes was not violated. No outliers were found (all Cooks Distance > 1) in any of the multiple imputations sets. Tables 3 and 4 show the results of the analyses: the independent variables which were found to be associated with higher (3) and lower (4) odds, around age 7, of ASD + DB compared to ASD – DB, ASD + DB compared to controls, and ASD − DB compared to controls, with *n* (subset A/subset B) ASD + DB = 178/307, *n* ASD − DB = 120/198, and *n* controls = 3,385/5,896. A variable was assumed to be associated if it had a non-zero *b*-coefficient in the lasso analysis in at least six out of ten multiple imputation sets in subset A and subset B (if included in B). To give an estimate of the magnitude of associations, the range (min–max) of odds ratios (*e*^*b*^) across the multiple imputation sets of subset A and B together are displayed per variable. Odds ratios should be interpreted with caution since lasso creates an upward bias for non-zero coefficients, and thus also for the converted odds ratios. See Online Resource Table 1 for the scale range of the continuous variables. Online Resource Table 5 displays the range and number of non-zero *b*-coefficients (i.e., change in log odds) per variable across the ten multiple imputation sets of subsets A and B, for each group comparison, and the range of the penalty terms (lambda) used in the lasso analyses.

**Table 3:** Lasso analyses: independent variables associated with higher odds of ASD + DB versus ASD − DB, ASD + DB versus controls, and ASD − DB versus controls, with the range of odds ratios across the multiple imputation sets of subset A (all variables; participants with few missing values) and B (variables with few missing values; all participants)

Variable category	ASD + DB versus ASD − DB		ASD + DB versus controls		ASD – DB versus controls	
	Higher odds of ASD + DB		Higher odds of ASD + DB		Higher odds of ASD − DB	
	Variable	Range OR	Variable	Range OR	Variable	Range OR
Family demographics	Partner’s medium social class (reference = high)	1.001–1.411	Maternal low social class (reference = high)	1.043–1.704		
	Partner’s vocational education level (reference = CSE/none)	1.007–1.525				
			Number of child’s siblings 18 months	1.002–1.160		
Pregnancy variables	Smoking mother	1.013–1.370	Smoking mother	1.103–1.240		
	Smoking partner	1.007–1.309	Smoking partner	1.029–1.229		
	Maternal antidepressant use	1.171–3.241				
			Infections in second trimester	1.019–1.115		
			Prenatal stress (life events)	1.009–1.024		
			Premature birth	1.001–1.237		
			High level of street traffic	1.040–1.219		
Delivery variables	Breech presentation at delivery/cesarean section*	1.116–2.846				
Neonatal variables	Feeding problems*	1.054–2.257				
Child variables	Male sex	1.290–1.562	Male sex	2.199–2.532	Male sex	1.121–1.368
	Temperament: adaptability (difficulty with waiting or change)	1.015–1.067	Temperament: adaptability (difficulty with waiting or change)	1.043–1.075		
	Temperament: intensity (reacts strongly)	1.038–1.061	Temperament: intensity (reacts strongly)	1.049–1.064		
	Temperament: persistence (reduced attention span)	1.005–1.026	Temperament: persistence (reduced attention span)	1.024–1.038		
			Temperament: activity	1.026–1.054		
					Temperament: mood (negative mood)	1.005–1.040
Preschool parental and family variables	Negativity scale score 47 months mother	1.034–1.068	Negativity scale score 47 months mother	1.230–1.273	Negativity scale score 47 months mother	1.147–1.192
	Negativity scale score 47 months partner*	1.001–1.073	Negativity scale score 47 months partner*	1.137–1.203	Negativity scale score 47 months partner*	1.031–1.095
					Positivity scale score 47 months partner*	1.046–1.214
			Parental bonding 8 months partner	1.007–1.039		
	Maternal antisocial behavior	1.001–1.099	Maternal antisocial behavior	1.091–1.156		
			Child maltreatment	1.171–1.297	Child maltreatment	1.065–1.206
			Aggression between parents 8 months (mother-rated)	1.002–1.052	Rows between parents 33 months (partner-rated)*	1.028–1.057
			Maternal depression	1.003–1.023	Maternal depression	1.013–1.040
					Partner’s anxiety	1.010–1.050
					Stress (life events)	1.002–1.013
					Accident prevention measures (in household)	1.013–1.074

A variable is only displayed if it had a non-zero *b*-coefficient in at least six out of ten multiple imputation sets in subset A (*n* = 3,683, *n* ASD + DB = 178, *n* ASD − DB = 120, *n* controls = 3,385; 100 variables) and subset B (*n* = 6,401, *n* ASD + DB = 307, *n* ASD − DB = 198, *n* controls = 5,896; 79/100 variables), or, for variables only included in subset A, had a non-zero *b*-coefficient in at least six out of ten multiple imputation sets in subset A. Qualitatively, presence of six or more non-zero regression coefficients across the multiple imputation sets was used to indicate association of an independent variable. Quantitatively, the range of odds ratios (lowest-highest OR) across multiple imputations is displayed as outcome. The odds ratio indicates the increase of odds when a variable increases with one, either from absence to presence, or on a continuous scale (see Online Resource Table 1 for the range of the scale). Odds ratios should be interpreted with caution since lasso creates an upward bias for non-zero *b*-coefficients, and thus also for converted odds ratios

*Variable only included in subset A

*Education* Vocational level, O level, A level, and degree in comparison with reference = CSE level/no education. *Social class* medium and low social class in comparison with reference = high social class. *Positivity scale score* degree of positive relationship between parent and child. *Parental bonding* degree of parental enjoyment and confidence regarding parenting and child. *Parenting score* frequency with which parents do activities with child. *Positive parenting experience* degree to which parents experience being a parent positively

**Table 4:** Lasso analyses: independent variables associated with lower odds of ASD + DB versus ASD − DB, ASD + DB versus controls, and ASD − DB versus controls, with the range of odds ratios across the multiple imputation sets of subset A (all variables in participants with few missing values) and B (variables with few missing values in all participants)

Variable category	ASD + DB versus ASD – DB		ASD + DB versus controls		ASD – DB versus controls	
	Lower odds of ASD + DB		Lower odds of ASD + DB		Lower odds of ASD – DB	
	Variable	Range OR	Variable	Range OR	Variable	Range OR
Family demographics	Summer birth	0.715–0.836				
			Twin birth	0.474–0.743		
Pregnancy variables	Weekly seafood consumption	0.733–0.971	Weekly seafood consumption	0.662–0.885		
	Diabetes	0.499–0.908				
			Vaginal bleeding in pregnancy	0.772–0.957		
Delivery variables					Breech presentation before labor*	0.816–0.997
Neonatal variables	Anemia	0.750–0.950	Anemia	0.754–0.907		
					Feeding problems*	0.760–0.903
Child variables	Temperament: approach (difficulty with the unknown)	0.981–0.999	Temperament: approach (difficulty with the unknown)	0.989–0.997		
	Temperament: threshold (quicker to notice things)	0.948–0.967	Temperament: threshold (quicker to notice things)	0.944–0.964		
			Intelligence	0.978–0.993	Intelligence	0.986–0.997
Preschool parental and family variables	Positivity scale score 47 months mother	0.880–0.929	Positivity scale score 47 months mother	0.815–0.891		
	Positivity scale score 47 months partner*	0.762–0.895	Positivity scale score 47 months partner*	0.891–0.994		
	Parenting score 6 months mother	0.833–0.882	Parenting score 18 months partner	0.983–0.997		
	Parental bonding 33 months mother	0.966–0.987	Parental bonding 33 months mother	0.924–0.957	Parental bonding 33 months mother	0.973–0.991
			Warmth between parents 33 months (mother-rated)	0.972–0.992	Warmth between parents 33 months (mother-rated)	0.989–0.999
					Affection between parents 8 months (partner-rated)*	0.984–0.999
	Accident prevention measures (in household)	0.914–0.995				

A variable is only displayed if it had a non-zero *b*-coefficient in at least six out of ten multiple imputation sets in subset A (*n* = 3,683, *n* ASD + DB = 178, *n* ASD − DB = 120, *n* controls = 3,385; 100 variables) and subset B (*n* = 6,401, *n* ASD + DB = 307, *n* ASD − DB = 198, *n* controls = 5,896; 79/100 variables), or, for variables only included in subset A, had a non-zero *b*-coefficient in at least six out of ten multiple imputation sets in subset A. Qualitatively, presence of six or more non-zero regression coefficients across the multiple imputation sets was used to indicate association of an independent variable. Quantitatively, the range of odds ratios (lowest-highest OR) across multiple imputations is displayed as outcome. The odds ratio indicates the increase of odds when a variable increases with one, either from absence to presence, or on a continuous scale (see Online Resource Table 1 for the range of the scale). Odds ratios should be interpreted with caution since lasso creates an upward bias for non-zero *b*-coefficients, and thus also for converted odds ratios

*Variable only included in subset A

*Education* Vocational level, O level, A level, and degree in comparison with reference = CSE level/no education. *Social class* medium and low social class in comparison with reference = high social class. *Positivity scale score* degree of positive relationship between parent and child. *Parental bonding* degree of parental enjoyment and confidence regarding parenting and child. *Parenting score* frequency with which parents do activities with child. *Positive parenting experience* degree to which parents experience being a parent positively

### Explained Deviance

The results of the ASD + DB versus ASD – DB model showed that explained deviance, i.e., the total fit of the model, ranged from 22.5 to 30.6% in subset A (*n* = 298) and from 16.0 to 19.6% in subset B (*n* = 505) across the ten imputation sets. In the ASD + DB versus controls model, independent variables explained 33.1 to 35.1% of deviance in subset A (*n* = 3,385) and 29.2 to 31.2% in subset B (*n* = 6,203). The ASD – DB versus controls model explained 7.6 to 12.4% of deviance in subset A (*n* = 3,505) and 6.6 to 8.7% in subset B (*n* = 6,094). Table 5 shows the percentage of explained deviance per variable category in each group comparison in subset A. Of note, these percentages do not add up to the explained deviance in the total model due to interrelationship of variables (i.e., leaving out one variable category can lead to other variable categories taking over explained deviance) and should thus be seen as estimations.

**Table 5:** Mean explained deviance in percentages per variable category in the models comparing ASD + DB versus ASD − DB, ASD + DB versus controls, and ASD − DB versus controls, across the ten imputation sets in subset A

Variable category	ASD + DB vs. ASD − DB	ASD + DB vs. controls	ASD – DB vs. controls
Family demographics	1.39	0.81	0.12
Pregnancy	3.59	1.13	0.49
Delivery	0.76	0.11	0.65
Neonatal	0.68	0.11	0.17
Child	5.60	7.45	1.67
Preschool parental and family	6.32	11.66	5.33

Subset A *n* ASD + DB = 178, *n* ASD – DB = 120, *n* controls = 3,385; 100 variables

### Family Demographics

Medium social class and vocational education level during pregnancy, both of the mother’s partner, were associated with higher odds of ASD + DB around age 7 in comparison to ASD – DB. Summer birth was associated with lower odds of being in the ASD + DB group versus the ASD − DB group. A lower maternal social class during pregnancy and a higher number of child’s siblings at 18 months were found to be associated with higher odds of ASD + DB versus controls, while twin birth was associated with lower odds of ASD + DB versus controls.

### Pregnancy, Delivery, and Neonatal Variables

With regard to pregnancy variables, smoking during pregnancy (of the child’s mother and her partner) was associated with higher odds of ASD + DB around age 7 versus ASD − DB and versus controls. Eating seafood on a weekly basis was associated with lower odds of ASD + DB versus ASD – DB and versus controls. Maternal antidepressant use was associated with higher odds of ASD + DB compared to ASD − DB. In contrast, maternal diabetes was associated with lower odds of ASD + DB versus ASD − DB. Maternal infections, prenatal stress (life events), a high level of street traffic, and being born prematurely were associated with higher odds of ASD + DB versus controls, whereas vaginal bleeding was associated with lower odds of ASD + DB versus controls.

Of the measured delivery and neonatal variables, a breech presentation and feeding problems in the first two weeks after birth were associated with higher odds of ASD + DB versus ASD – DB, whereas breech presentation and feeding problems were associated with lower odds of ASD − DB versus controls. Neonatal anemia was associated with lower odds of ASD + DB versus ASD − DB and versus controls.

### Child Variables

Male sex was associated with higher odds of ASD + DB versus ASD − DB around age 7, and versus controls, but also with higher odds of ASD − DB versus controls. Of preschool temperament characteristics, measured at 24 months, a temperament characterized by less difficulty with the unknown, being less quick to notice things, more difficulty with waiting or change, stronger reactions, and a reduced attention span were all associated with higher odds of ASD + DB versus ASD − DB and versus controls around age 7. In addition, a temperament characterized by more activity was related to higher odds of ASD + DB versus controls. In contrast, a temperament characterized by a more negative mood was associated with higher odds of ASD − DB versus controls. Higher intelligence was associated with lower odds of ASD + DB versus controls and with lower odds of ASD − DB versus controls.

### Preschool Parental and Family Variables

Of the preschool parental and family variables, a more negative parent–child relationship (i.e., negativity scale of mother as well as her partner) and maternal antisocial behavior were associated with higher odds of ASD + DB around age 7 versus ASD − DB and versus controls. A more positive parent–child relationship (i.e., positivity scale), a higher degree of maternal enjoyment and confidence (bonding), and a higher frequency of parent–child activities (parenting score) were associated with lower odds of ASD + DB versus ASD − DB and versus controls. A more negative parent–child relationship was also associated with higher odds of ASD − DB versus controls, while maternal bonding was also associated with lower odds of ASD − DB versus controls. The use of more accident prevention measures in the household was associated with lower odds of ASD + DB versus ASD − DB and with higher odds of ASD – DB versus controls.

A worse psychological health of parents in the child’s preschool years, a less optimal relationship between parents (i.e., more aggression/less affection), and more child maltreatment were associated with higher odds of ASD + DB around age 7 versus controls and with higher odds of ASD − DB versus controls. Stress during the child’s preschool years (life events) was associated with higher odds of ASD − DB versus controls. In comparison to controls, there were also positive parent–child factors associated with higher odds of ASD + DB and ASD − DB, i.e., mother’s partner’s bonding to the child was associated with higher odds of ASD + DB and a more positive relationship between partner and child was associated with higher odds of ASD − DB.

## Discussion

We examined the association of a wide range of early-life factors up to age 4 with the presence of disruptive behaviors in children with autistic traits around age 7. Investigation of factors that discriminate between children with autistic traits *with* versus *without* disruptive behaviors, indicated that maternal antidepressant use, prenatal smoking, not consuming seafood during pregnancy, a low prenatal social economic situation, a breech presentation at delivery, neonatal feeding problems, child male sex, child difficult temperament, a less optimal preschool family environment, and maternal antisocial behavior were all associated with the presence of disruptive behaviors in children with autistic traits.

We found that many of above early-life factors were only associated with the presence of disruptive behaviors in children with autistic traits, but not with the presence of autistic traits without disruptive behaviors in comparison to unaffected controls (i.e., children without autistic traits and no disruptive behaviors). This was also apparent from the estimated total level of fit of the tested models, that lay around 20% for the ASD + DB versus ASD − DB model, but around 10% for the ASD – DB versus controls model.

Prenatal parental smoking was one variable that was associated with the presence of disruptive behaviors in children with autistic traits, but not with the presence of autistic traits without disruptive behaviors, a finding that is in line with other studies that did not find an association between smoking and ASD (Rosen et al., 2015; Wang et al., 2017). A study that did point to a relation between prenatal smoking and ASD (St. Pourcain et al., 2011) included children with ASD, who mainly also had externalizing problems. It may thus be that the association in that study stemmed from an association between smoking and ASD with co-occurring disruptive behaviors. Also regarding child temperament, measured at 24 months, we found many aspects (e.g., impaired adaptability, intensity, persistency, and approach) to be associated with the presence of disruptive behaviors in children with autistic traits around age 7, while only one temperamental aspect (i.e., a more negative mood) was associated with the presence of autistic traits without disruptive behaviors in comparison to unaffected controls. Our findings are in in line with studies that suggest that early temperament may be a vulnerability for later disruptive behavior (Nigg, 2006; Rijlaarsdam et al., 2016).

Just as prenatal smoking and a difficult child temperament, not consuming seafood during pregnancy, a breech presentation at delivery, neonatal feeding problems, and a low prenatal social economic situation were related to the presence of disruptive behaviors in seven-year-old children with autistic traits, but not to the presence of autistic traits without disruptive behaviors. In addition, in comparison to unaffected controls, maternal infections during pregnancy, traffic related air pollutants, premature birth, and a large family size were associated with autistic traits with disruptive behaviors, but not with autistic traits without disruptive behaviors. This finding thus also indicates a specific association of these factors with ASD *with* disruptive behaviors and not *without*, even though no effect was found in comparison of the two ASD groups themselves. Our findings on low social economic situation, large family size, and pregnancy related complications correspond to studies in which these factors were associated with disruptive behaviors in ASD (Chandler et al., 2016; Gadow et al., 2008a; Midouhas et al., 2013). In other studies, however, above factors have been associated with ASD (Bölte et al., 2019; Emberti Gialloreti et al., 2019; Gardener et al., 2011; Julvez et al., 2016; Wang et al., 2017). Given our findings, it may be that findings in previous studies on ASD were driven by co-occurring disruptive behaviors.

In contrast to the factors described above, other factors were associated with the presence of disruptive behaviors in children with autistic traits, but also with the presence of autistic traits without disruptive behaviors in comparison to unaffected controls: child male sex, maternal depression, and a less optimal preschool family environment. Yet, in previous studies (Brereton et al., 2006; Chandler et al., 2016; Simonoff et al., 2008), male sex was not found to be related to disruptive behaviors in ASD. The high number of girls in our ASD groups (about 33% versus less than 20% in other studies) may have provided more room for finding sex differences. This high number of girls may stem from defining the ASD groups based on high levels of autistic traits, instead of a clinical diagnosis. Since girls with ASD are less likely to receive a diagnosis than boys (Lai & Baron-Cohen, 2015), our results emphasize the value of studying ASD as the extreme of an autistic trait dimension (Constantino & Charman, 2016; Robinson et al., 2011; Ronald & Hoekstra, 2011). The association that we found between male sex and autistic traits without disruptive behaviors is in line with previous studies into ASD (Baio et al., 2018; St. Pourcain et al., 2011).

Regarding maternal depression, we found that maternal prenatal antidepressant use was related to the presence of disruptive behaviors in children with autistic traits and that maternal depressive symptoms were associated with the presence of autistic traits without disruptive behaviors in comparison to controls. Since the role of antidepressants can be (partially) explained through maternal mental illness (Andrade, 2017; Rai et al., 2017; Rotem-Kohavi & Oberlander, 2017), our results suggest that maternal depression may be related to ASD and, above that (shown through the association of antidepressants), to disruptive behaviors in children with ASD. This is in line with other studies showing parental psychopathology to be related to disruptive behaviors in ASD (Gadow et al., 2008a; McRae et al., 2019) and with studies indicating no direct effect of antidepressants on externalizing behavior (see Rotem-Kohavi & Oberlander, 2017).

Last, a suboptimal preschool family environment was also associated with the presence of disruptive behaviors in children with autistic traits around age 7 as well as with the presence of autistic traits without disruptive behaviors compared to controls. Of preschool family environment factors, fewer parent–child activities and fewer household accident prevention measures were specifically associated with the presence of disruptive behaviors in children with autistic traits, while a worse parent–child relationship, and less parental enjoyment and confidence in parenting were associated with disruptive behaviors in children with autistic traits, but also with autistic traits without disruptive behaviors. These findings are in line with other studies that suggest an association between a less optimal early parent–child relationship and disruptive behaviors in children with ASD (Maljaars et al., 2014; McRae et al., 2019; Midouhas et al., 2013), as well as with ASD (Mandy & Lai, 2016). Our results indicate that a worse family environment during the preschool period is associated with the presence of autistic traits *with* and *without* disruptive behaviors at child’s later age, but that this association may apply more strongly to autistic traits *with* disruptive behaviors.

In this study, we did not look into factors associated with the presence of disruptive behaviors in children *without* autistic traits. This was because our focus was on children with autistic traits: adding a fourth ‘DB-only’ group would make the manuscript lose its focus. Furthermore, already quite some studies have been done on factors associated with disruptive behaviors in non-ASD children. When comparing our findings on factors associated with the presence of disruptive behaviors in children with autistic traits to the literature on factors associated with disruptive behaviors in non-ASD populations, it is interesting to see that many factors coincide. This is consistent with previous studies that suggest similarities in factors associated with disruptive behaviors in ASD and non-ASD populations (Gadow et al., 2008b; Mandy et al., 2014). Coinciding factors include prenatal smoking (Dolan et al., 2016; Latimer et al., 2012; Ruisch, Buitelaar, et al., 2018; Ruisch, Dietrich, et al., 2018), low SES (Carneiro et al., 2016; Ruisch, Buitelaar, et al., 2018), maternal antisocial behavior (Davies et al., 2012), maternal depression (Guney et al., 2015), a less optimal preschool family environment (Gach et al., 2018), child male sex (Carneiro et al., 2016), and child difficult temperament (Bao et al., 2016; Carneiro et al., 2016).

Finally, our results showed that a few child and environmental factors were related to both ASD groups (with and without disruptive behaviors) in comparison to unaffected controls. These were lower child intelligence, preschool child maltreatment, and a bad relationship between parents. Furthermore, stress, which was measured by number and impact of life events, was also associated with both ASD groups, albeit at a different age: stress during pregnancy was associated with the presence of autistic traits with disruptive behaviors around age 7, while environmental stress during the preschool period was associated with the presence of autistic traits without disruptive behaviors. The period in which stress occurs may thus be important. In another study, maternal stress during pregnancy was not associated with ASD, in contrast to stress during preschool years (Rai et al., 2012). In line with our results on prenatal stress, maternal stress during pregnancy has also been related to externalizing behavior in non-ASD populations (Latimer et al., 2012; MacKinnon et al., 2018; Ruisch, Buitelaar, et al., 2018).

Overall, our results show that some early-life environmental and child factors in our study are associated with autistic traits regardless of the presence of disruptive behaviors around age 7, but that more of the studied early-life and child factors are related to autistic traits *with* disruptive behaviors. The factors that were related to the presence of disruptive behaviors in children with autistic traits came from all investigated variable categories: family demographics, pregnancy, delivery, neonatal, child, and preschool parental and family factors. Our results suggest that pregnancy, child, and preschool parental and family factors may be especially related to the presence of disruptive behaviors in children with autistic traits around age 7. With regard to the clinical implications of our study, our results indicate that it is important for clinicians and parents to be aware that early-life environmental and child factors may be associated with the presence of disruptive behaviors in a child with autistic traits or ASD at a later age.

### Strengths and Limitations

Strengths of our study include the prospective measurement of a comprehensive range of factors (thus eliminating recall bias) in a large population sample. The longitudinal design of ALSPAC provided us with the opportunity to examine the association of early-life environmental factors with autistic traits and disruptive behaviors at a later age, i.e., around age 7. However, we cannot draw conclusions about the associations of these factors with disruptive behaviors and autistic traits measured closer to the age at which the factors were measured. Future research may look into these concurrent associations as well as the possible reciprocity of relationships of the studied factors with disruptive behaviors and autistic traits.

Defining our ASD groups by means of an autistic traits cut-off, instead of a clinical diagnosis, may be seen as a limitation. However, our SCDC cut-off has adequate discriminative value for ASD (Skuse et al., 2005), and we therefore think that our findings may be extended to children with a clinical ASD diagnosis. Furthermore, using a traits cut-off may also have advantages, leading to inclusion of children who are less likely to receive a clinical diagnosis, for example girls (Constantino & Charman, 2016). In future research it would be interesting to see how the variables found in relation to autistic traits hold when studied in children with a clinical diagnosis of ASD.

Another limitation is that, even with many variables included, our study may have overlooked variables of interest for ASD or disruptive behavior, e.g., prenatal maternal valproate use, nutrition, substance use, and parenting style. Furthermore, our study design (i.e., lasso analyses) was chosen to indicate whether a specific variable was or was not associated, and thus not to indicate the strength of individual associations. Future research may look further into the strength of the associations found in the current study.

Finally, the ASD + DB group scored higher on autistic traits than the ASD − DB group and the ASD − DB group scored higher on disruptive behaviors than the control group. The associations with regard to the presence of disruptive behaviors in children with autistic traits may thus partly stem from higher levels of autistic traits in the ASD + DB group, although, for most associated factors this does not seem likely since these factors were not associated with the presence of autistic traits without disruptive behaviors. Future research could aim to more thoroughly disentangle the effects of more severe autistic traits from co-occurring disruptive behaviors.

## Conclusion

A multitude of early-life environmental and child factors appears to be associated with the presence of disruptive behaviors in seven-year-old children with autistic traits, i.e., prenatal smoking, not consuming seafood during pregnancy, a breech presentation at delivery, neonatal feeding problems, a low social economic situation, child difficult temperament, child male sex, a less optimal preschool family environment, maternal depression, and maternal antisocial behavior. In addition, our results indicate that child male sex, maternal depression, and a less optimal preschool family environment are also related to the presence of autistic traits without disruptive behaviors. Overall, early-life factors may thus be more related to ASD with disruptive behaviors than to ASD without disruptive behaviors, demonstrating the importance of distinguishing between groups of children with ASD to improve understanding of the association of early-life factors with ASD and disruptive behaviors at a later age.

## Supplementary Information

Below is the link to the electronic supplementary material.

Supplementary file1 (DOCX 57 kb)

Supplementary file2 (DOCX 18 kb)

Supplementary file3 (DOCX 18 kb)

Supplementary file4 (DOCX 18 kb)

Supplementary file5 (DOCX 76 kb)
