# Supplementary material for: Early-Life Environmental and Child Factors Associated with the Presence of Disruptive Behaviors in Seven-Year-Old Children with Autistic Traits in the Avon Longitudinal Study of Parents and Children
Source: J Autism Dev Disord. 2021 Jul 10;52(6):2747–61. doi: 10.1007/s10803-021-05081-x (PMC9114014; doi:10.1007/s10803-021-05081-x)
Supplement: Supplementary file 1 — Supplementary file1 (DOCX 57 kb) [file 10803_2021_5081_MOESM1_ESM.docx]

**Online Resource Table 1** Overview of independent variables: data collection, description, and usage

| **Variable** | **Measurement moment** | **Data collection in ALSPAC** | **Measurement scale** | **Description** | **Definition of missings** |
| --- | --- | --- | --- | --- | --- |
| **Family demographics** | | | | | |
| *Parental age*  Maternal age at time of birth  High partner’s age at time of birth | 8 weeks  8 weeks | Both parents were asked for their date of birth. | Ordinal  Dichotomous | From the date of birth, ALSPAC derived age in years at birth.  Low (< 20), Middle (20 - 29), or High (> 29).  Low/Middle (< 35), or High (≥ 35). | Both variables were categorized after multiple imputation of age in years. |
| Firstborn child | 18 weeks gestation | Mother answered questions about the number of previous pregnancies, miscarriages and abortions. | Dichotomous | Being pregnant of a firstborn. From mothers’ answers, ALSPAC derived the number of previous pregnancies resulting in livebirth or stillbirth. We assumed being pregnant of a firstborn if this number was zero. | Missing if no answer was given on miscarriages as well as abortions. |
| Summer birth |  | Month of birth was recorded. | Dichotomous | Summer birth comprised births in June, July, and August. | Dichotomized after multiple imputation of a variable indicating season of birth. |
| Twin birth |  | Clinical records. | Dichotomous | A pregnancy resulting in a twin delivery. |  |
| *Social Economic Status*  Maternal education  Partner’s education  Maternal social class  Partner’s social class | 32 weeks gestation  32 weeks gestation  32 weeks gestation  32 weeks gestation | Mother was asked what educational qualifications she and her partner had.  Mother was asked to indicate her own occupation and that of her partner. | Ordinal  Ordinal  Ordinal  Ordinal | Educational qualifications were categorized in levels: CSE (certificate of secondary education or general certificate of secondary education [GCSE] D - G) / none, Vocational, O level (Ordinary level or GCSE A - C), A level (Advanced level), and Degree (university degree).  Three social class levels were computed by dividing ALSPAC’s six levels into High (‘Professional’ and ‘Managerial and technical’), Medium (‘Skilled non-manual’ and ‘Skilled manual’), and Low (‘Partly skilled’ and ‘Unskilled’). | If no answer was given, CSE/none was assumed. |
| *Family composition*  Single household at 8 months  Single household at 21 months  Single household at 33 months  Single household at 47 months  Number of child’s siblings 18 months  Number of child’s siblings 30 months | 8 months  21 months  33 months  47 months  18 and 47 months  30 and 47 months | Mother was asked if she had a partner who lived with her.  At 18 and 30 months mother was asked about the number of child’s younger siblings. At 47 months she was asked about the number of older siblings. | Dichotomous  Dichotomous  Dichotomous  Dichotomous  Ratio  Ratio | These scores indicated whether the study mother lived without a partner at different measurement moments.  Estimation of the number of siblings at 18 and 30 months. We derived the scores by adding the number of older siblings to the number of younger siblings at 18 and 30 months, respectively. | A score was rated missing if information at one or more measurement moments was missing. |
| **Pregnancy** | | | | | |
| *Infections*  Infections in first trimester  Infections in second trimester  Infections in third trimester | 18 weeks gestation  32 weeks gestation  8 weeks | Mother indicated if each of a list of infections was present during each of the three trimesters. | Dichotomous  Dichotomous  Dichotomous | Any of the following occurred per trimester: urinary infection, influenza, rubella, thrush (candida), genital herpes, or any other infection. | For each trimester, the infections score was rated missing when all infection items of that measurement were missing. |
| *Substance use*  Maternal alcohol use  Smoking mother  Smoking partner | Pregnancy  Pregnancy  Pregnancy | In the first (categorical), second (glasses per week) and third (categorical) trimester, mother indicated how much she had to drink.  At each trimester mother indicated how many cigarettes she and her partner (only at first and third trimester) had smoked per day. | Dichotomous  Dichotomous  Dichotomous | Maternal consumption of any alcohol in pregnancy, computed by aggregating the scores from the first, second, and third trimester.  Any smoking by mother and her partner in pregnancy, computed by aggregating the scores from the first, second (maternal only), and third trimester. | Rated missing if alcohol scores were missing in all three trimesters.  Scores were computed after multiple imputation of the smoking scores per trimester. |
| *Medication*  Maternal medication use  Maternal antidepressant use | Pregnancy  Pregnancy | In the first and second trimester, mother was asked to write down all pills, medicines, and ointments that she used. Coders added the number of different items.  In the first and second trimester, mother was asked to indicate if she had taken medication for depression. | Dichotomous  Dichotomous | Any maternal medication use as computed by aggregating the scores from the first and second trimester.  Any maternal antidepressant use as computed by aggregating the scores from the first and second trimester. | Rated missing if medication use scores were missing in both trimesters.  The score was computed after multiple imputation of the antidepressant score in the first and second trimester. |
| Diabetes | Pregnancy | At recruitment, mother was asked about existing diabetes and any previous history of gestational diabetes. Current gestational diabetes and glycosuria were abstracted from medical records. | Dichotomous | ALSPAC created four groups: No glycosuria or diabetes, Existing diabetes, Gestational diabetes, and Glycosuria.  We dichotomized these groups to indicate any evidence of diabetes present during pregnancy. |  |

| Maternal pre-pregnancy weight | 12 weeks gestation | Mother was asked what her weight was before her pregnancy started. | Ratio | Maternal weight in kg before pregnancy. |  |
| --- | --- | --- | --- | --- | --- |
| High level of street traffic | 8 months | Mother was asked how she would rate the level of traffic in her street, on a 5-point scale. | Dichotomous | A ‘busy’ or ‘very busy’ street, versus a ‘very quiet’, ‘quiet’ or ‘moderate’ street. |  |
| *Nutrition*  Weekly seafood consumption  Folic acid intake | 32 weeks gestation  18 weeks gestation | Mother was asked to indicate how many times she ate three kinds of (shell)fish. Answer options ranged from ‘never/rarely’ to ‘more than once a day’.  Mother was asked if she had been taking folic acid/folate during pregnancy. | Dichotomous  Dichotomous | Consumption of white, oily and/or shellfish at least once a week, computed by aggregating the scores on the three (shell)fish questions.  Intake of folic acid during pregnancy. |  |
| *Mothers’ mental health*  Maternal prenatal anxiety  Maternal prenatal depression  Prenatal stress  Maternal external locus of control | 18 weeks gestation  18 weeks gestation  Pregnancy  12 weeks gestation | Measured with the items of the Crown-Crisp Experiential Index, Anxiety and Depression subscale (Crown & Crisp, 1997, in ALSPAC).  In the first and second half of pregnancy, mother was asked to indicate if any of 41 ‘events which may have brought changes in your life’ had occurred (e.g. a death, illness, problems or changes regarding work, moving, arguing with partner or family, and pregnancy related incidents) and how much she had been affected per occurred event.  Measured with the Adult locus of control scale (Nowicki & Strickland, see ALSPAC). Questions covered feeling that ‘it doesn’t pay off to try hard’ and believing that ‘bad things are going to happen no matter what you try’. | Interval  Interval  Interval  Interval | A higher score (range 0-16) indicated more anxiety or more depressive symptoms, respectively.  For the first and second pregnancy half, ALSPAC created a weighed life event score by summing the event ratings, with possible answers: event did not occur (0), did occur but did not affect (1), occurred and affected mildly (2), moderately (3), or a lot (4). We calculated the Prenatal stress score by averaging both weighted life event scores. A higher score (range 0-164) was assumed to indicate more stress.  A higher score (range 0-12) indicated feeling less in control/a more external locus of control. | A missing subscale item was replaced by the item’s mode, unless all items of the subscale were missing.  If an item (event) was missing, it was assumed it hadn’t occurred, unless all items of a measurement were missing. If all items of a measurement were missing, the weighted life event score from the other moment was used as Prenatal stress score.  If one or more items were missing, the mode of the missing item was used. |
| *Interpersonal relations*  Affection between parents  Aggression between parents | 12 weeks gestation  12 weeks gestation | Mother rated. ALSPAC measure based on work of Quinton and Rutter, and Stanley (see ALSPAC). Affection subscale items comprised listening, talking, and behaving affectionately towards each other. Aggression subscale items comprised getting angry at each other and having arguments. | Interval  Interval | ALSPAC computed subscale scores were reversed such that a higher affection score (range 6-30) indicated more affection between parents and a higher aggression score (range 3-15) indicated more aggression. | A missing subscale item was replaced by the item’s mode, unless all items of that subscale were missing. |
| Maternal non-positive pregnancy feelings | 18 weeks gestation | Mother was asked: ‘How do you feel about your pregnancy now?’. Answer options were: overjoyed, pleased, mixed feelings, not happy, very unhappy, no particular feelings. | Dichotomous | Feeling ‘mixed’, ‘indifferent’, ‘unhappy’ or ‘very unhappy’, versus feeling ‘overjoyed’ or ‘pleased’. | The dichotomized score was computed after multiple imputation of the score including all six categories. |
| Pre-eclampsia | Pregnancy | Clinical records. | Dichotomous | Occurrence of pre-eclampsia during pregnancy. |  |
| Vaginal bleeding in pregnancy | Pregnancy | At 18 weeks and 32 weeks gestation, and 8 weeks mother was asked to indicate if vaginal bleeding had occurred. Furthermore, information was abstracted from clinical records. | Dichotomous | Any vaginal bleeding during pregnancy as computed by aggregating information from the three trimesters, using maternal information as well as clinical records. | This score was computed after multiple imputation of vaginal bleeding scores per trimester. Per trimester, a score was rated missing if no information was available from mother as well as clinical records. |
| Premature birth |  | Gestation length was abstracted from obstetric clinical records. | Dichotomous | Birth was rated premature if length was less than 37 weeks. |  |
| Low birthweight |  | Birth weight was abstracted from obstetric data, measurements or birth notification. | Dichotomous | Birth weight was rated low if less than 2500 grams. |  |
| **Delivery** | | | | | |
| *Breech presentation before labor  *Breech presentation at onset of labor  *Breech presentation at delivery / cesarean section  *Cesarean section  *Breech birth |  | Clinical records.  Information was collected on presentation (breech, vertex or other presentation) at three moments.  Information was collected on the occurrence of a cesarean section and its nature (elective or emergency). | Dichotomous  Dichotomous  Dichotomous  Dichotomous  Dichotomous | Breech presentation versus vertex or other.  Cesarean section  (either elective or emergency) versus no cesarean section.  Breech birth indicated a birth (not a cesarean section) in combination with a breech presentation at delivery. | The variable cesarean section was dichotomized after multiple imputation of the variable with categories no cesarean section, elective, or emergency.  The variable was computed from ‘Breech presentation at delivery/ cesarean section’ and ‘Cesarean section’. |
| *Maternal hemorrhage prior to delivery  *Precipitate labor  *Umbilical cord complications |  | Clinical records.  Information was collected on maternal hemorrhage in labor or at cesarean section, and its reason.  Information was collected on the notion of cord around the baby’s neck and the notion of a cord prolapse. | Dichotomous  Dichotomous  Dichotomous | Maternal hemorrhage in labor or cesarean section (either placenta previa, abruption or unspecified).  Precipitate labor noted.  Any umbilical cord complication noted in labor or at cesarean section. This variable was computed by aggregating the information on both kinds of cord complications. | Rated missing if there was no information on both kinds of cord complications. |
| **Neonatal** | | | | | |
| *Low Apgar at 5 minutes | 5 minutes | Clinical records.  Information was collected on Apgar score (range 0-10). | Dichotomous | Apgar score 0 through 6 (low), versus 7 through 10. |  |
| *Child’s age at discharge from hospital |  | Clinical records.  Measured in days. | Ratio | Child’s age at discharge from hospital of birth in days with 0 being less than 24 hours. |  |
| Anemia  *Feeding problems  *Oxygen problems |  | Medical records.  Information was collected on the occurrence of resuscitation with a bag, mask, and oxygen, resuscitation by facial oxygen, and apneic attacks in the first two weeks. | Dichotomous  Dichotomous  Dichotomous | Anemia noted in first two weeks.  Feeding problems noted in the first two weeks.  Any oxygen problems in the first two weeks. This score was computed by aggregating information on resuscitation with a bag, mask, and oxygen, resuscitation by facial oxygen, and the notion of apneic attacks. | Rated missing if there was no information available on both resuscitation methods and apneic attacks. |
| *Breastfeeding*  Breastfeeding in first four weeks  Duration of breastfeeding | 4 weeks  15 months | Mother was asked how she fed her baby during the first day and during each of the first four weeks.  Mother was asked if the baby had been breastfed, if he/she was still being breastfed, and if stopped, how old the baby was in months at that time. | Dichotomous  Ordinal | Any breastfeeding in the first four weeks, i.e. mother indicated that she had fed by breast (and bottle) during one or more of the time periods.  Duration of breast feeding: never, < 3 months, 3 - 5 months, or ≥ 6months. |  |
| **Child** | | | | | |
| Intelligence | 8 years | Intelligence was measured by means of the Wechsler Intelligence Scale for Children, third edition^UK^ (Wechsler, Golombok, & Rust, 1992, in ALSPAC). The 10 subtests, except for Coding, were administered in a shortened form. | Interval | Total intelligence. ALSPAC multiplied the scores on the shortened tasks to be able to calculate the age scaled scores. Total IQ was calculated using the WISC manual. | The score was rated missing if more than one subtest score was missing from either performance or verbal subtests. Age scaled scores were prorated if one performance and/or one verbal subtest was missing. |
| Non-white ethnicity | 32 weeks | Mother was asked how she described the race / ethnic group of herself and her partner. | Dichotomous | Non-white signified that mothers and/ or partners ethnic group was non-white. |  |
| Male sex |  |  | Dichotomous | Male sex |  |

| *Temperament*  Activity Score  Rhythmicity Score  Approach Score  Adaptability Score  Intensity Score  Mood Score  Persistence Score  Distractibility Score  Threshold Score | 24 months | Mother answered questions, which were adapted from the Carey Toddler Temperament Scale (Fullard, McDevitt, & Carey, 1984, in ALSPAC), on a 5 point rating scale. | Interval | Child temperament measured with 9 subscales. A higher score indicated, respectively: more activity (0-36), more variation in behavior (0-44), less approach (0-44), reduced ability to adapt (0-28), more intensity in reaction (0-36), more negative /less positive emotions (0-48), reduced attention span (0-36), higher distractibility (0-40), and being quicker to notice things (0-32). | A subscale score was rated missing if more than half of the scores of a subscale were missing. When less than half were missing, the subscale score was prorated. |
| --- | --- | --- | --- | --- | --- |
| **Preschool parental and family** | | | | | |
| *Interpersonal relations*  Mother’s experienced social support  Affection between parents, mother rated  Aggression between parents, mother rated  *Affection between parents, partner rated  *Aggression between parents, partner rated  Warmth between parents, mother rated  Rows between parents, mother rated  *Warmth between parents, partner rated  *Rows between parents, partner rated | 8 weeks - 21 months  8 months  8 months  8 months  8 months  33 months  33 months  33 months  33 months | At 8 weeks, 8 months, and 21 months mother rated an ALSPAC adapted questionnaire on social support (e.g. opportunity to share feelings, rely on others for help).  Parents rated an ALSPAC measure based on work of Quinton and Rutter, and Stanley (see ALSPAC). Subscales concerned affection (e.g. listening, talking, behaving affectionately) and aggression (e.g. getting angry at each other, having arguments).  Items of the Warmth subscale (part of the Intimate bond measure; Wilhelm & Parker, 1988, in ALSPAC) and Rows subscale (see ALSPAC) were similar to Affection and Aggression items at 8 months, respectively. | Interval  Interval  Interval  Interval  Interval  Interval  Interval  Interval  Interval | Mean experienced maternal social support. Computed by taking the mean score over the three measurement moments. A higher score (range 0-30) signaled a higher amount of experienced social support.  ALSPAC computed scores were reversed such that a higher affection score (range 10-50) indicated more affection between parents and a higher aggression score (range 3-15) indicated more aggression.    Scores were computed such that a higher score indicated more warmth (range 12-48) and rows (range 0-14), respectively. | The mean score was rated missing if all three measurements were missing. Items were replaced by their mode, unless all items of a measurement were missing.  A missing subscale (affection or aggression) item was replaced by the item’s mode, unless all items of that subscale were missing.  A subscale (warmth or rows) score was set to missing if any item of that subscale was missing. |
| *Parental psychology*  Maternal anxiety  Partner’s anxiety  Maternal depression  Partner’s depression  Stress  Maternal antisocial behavior | 8 weeks - 21 months  8 weeks - 21 months  8 weeks - 21 months  8 weeks - 21 months  21 and 33 months  12 years | At 8 weeks, 8 months, and 21 months parents rated the items of the Crown-Crisp Experiential Index, Anxiety and Depression subscale (Crown & Crisp, 1997, in ALSPAC).  At 21 and 33 months, mother was asked to indicate if any of 43 ‘events which may have brought changes in your life’ had occurred (e.g. a death, illness, problems or changes regarding work, moving, arguing with partner or family) and how much she had been affected per occurred event.  Mother was asked to indicate if 20 kinds of events/acts related to breaking the law had ever occurred (e.g. trouble with the law, driving with alcohol, stealing, using violence). | Interval  Interval  Interval  Interval  Interval  Ratio | Anxiety scores were calculated by taking the mean score of the anxiety subscale over the three measurement moments. Similarly, depression scores were calculated. A higher anxiety and depression score (range 0-16) indicated more anxiety and depressive symptoms, respectively.  For 21 and 33 months, ALSPAC created a weighed life event score by summing the event ratings, with possible answers: event did not occur (0), did occur but did not affect (1), occurred and affected mildly (2), moderately (3), or a lot (4).  We calculated the Stress score by averaging both weighted life event scores. A higher score (range 0-172) was assumed to indicate more stress.  The number of lifetime antisocial behaviors (range 0-20). | Anxiety and Depression scores were rated missing if scores of all its three measurement moments were missing. A missing subscale item was replaced by its mode, unless all items of that subscale were missing.  If an item (event) was missing, it was assumed it hadn’t occurred, unless all items of that measurement were missing. If all items of one of the measurement moments were missing, the score from the other moment was used.  If an item (event/act) was missing, it was assumed it hadn’t occurred, unless all items of that measurement were missing. The maternal antisocial behavior score was rated missing if all 20 items were missing. |
| *Parenting*  Accident prevention measures  Parenting score 6 months mother  Parenting score 6 months partner  Parenting score 18 months  mother  Parenting score 18 months partner  Parenting score 38 months mother  Parenting score 38 months partner  Parental bonding 8 months mother  *Parental bonding 8 months partner  Parental bonding 33 months mother  Positive parenting experience score mother  Negative parenting experience score mother  *Positive parenting experience score partner  *Negative parenting experience score partner    Positivity scale score mother  Negativity scale score mother  *Positivity scale score partner  *Negativity scale score partner | 8 months  6 months  6 months  18 months  18 months  38 months  38 months  8 months  8 months  33 months  21 months  21 months  21 months  21 months  47 months  47 months  47 months  47 months | Mother rated how many of six different safety measures she owned (e.g. safety gate, electric socket cover).  At 6, 18, and 38 months mother was asked questions on the frequency with which she and her partner did a range of activities with the child (e.g. playing, reading a book, and walking). Answer options varied from categorical answers (e.g. ‘often’), number of times per week, to yes/no.  Parents (partner only at 8 months) were asked to rate 11 items on how they felt about looking after their child on a four point rating scale, developed by ALSPAC.  Parents were asked how they felt about a series of opinions related to being a parent, on a four point rating scale.  Parents rated if statements about the relationship with their child applied to their situation. Positive items contained ‘I really love this child’ and ‘I feel very close to this child’. Negative items contained ‘I have frequent battles of will with this child’ and ‘I dislike the mess and noise that surrounds this child’. | Ratio  Interval  Interval  Interval  Interval  Interval  Interval  Interval  Interval  Interval  Interval  Interval  Interval  Interval  Interval  Interval  Interval  Interval | The number of different safety measures (range 0-6) present in the household.  The frequency of doing activities with the child at 6 months (range 0-14 for mother, 0-22 for partner), 18 months (range 0-51 for mother, 0-40 for partner) and 38 months (range 0-30 for both parents). A higher score indicated doing activities more often.  A higher score (range 0-33) indicated more parental enjoyment and confidence.  Scores were computed such that a higher positive experience score indicated feeling more positive toward the child (e.g. feelings of love and pleasure, range 5-20) and a higher negative score indicated feeling more negative (e.g. feeling angry and desperate, range 7-28).  Scale scores were created in correspondence with Dunn, Deater-Deckhard, Pickering, and Golding (1999). Answers were coded as yes, no, and both. A higher Positivity scale score and higher Negativity scale score indicated more agreement with positive items (range 0-8) and negative items (range 0-8), respectively. Items partly correspond to items of the Positive and Negative parenting experience scores. | A missing item was taken as an indication that an activity happened hardly ever. At 6 and 18 months, the parenting score was rated missing if every item was missing. At 38 months, the parenting score was rated missing when the first item was missing.  A missing item was replaced by its mode, unless all items were missing.  Parenting experience scores were rated missing if any subitem was missing.  A missing item was replaced by its mode, unless all items of a scale were missing. |
| Child maltreatment | 8 months - 47 months | Abuse: at 18, 30, and 42 months, mother was asked if any sexual and physical abuse had occurred. Furthermore, at 8, 21, and 33 months, mother was asked if she or her partner had been physically or emotionally cruel toward their child.  Maladaptive parenting: the study mother answered four questions on hitting (at 18, 21, 24, and 42 months), three questions on shouting (at 18, 24, and 42 months) and four questions on hostility (e.g. feeling hostile, for example irritated towards the child; at 21 and 47 months). | Dichotomous | Child maltreatment was deemed present when any abuse and/or severe maladaptive parenting had occurred in the child’s first three years.  Abuse and severe maladaptive parenting scores were calculated by means of summing and dichotomizing the scores on various parenting questions (i.e. done similarly by Lereya, Copeland, Costello, and Wolke [2015]).  Abuse was deemed present if questions concerning any sexual abuse, physical abuse, physical cruelty, or emotional cruelty were answered affirmative.  Severe maladaptive parenting was deemed present if all three kinds of maladaptive parenting had occurred: hitting, shouting, and hostility. Hitting was deemed present when it had happened at least often/ once a week at any of the measurements. Shouting was deemed present when it happened at least often/ every day at any of the measurements. Hostility was deemed present if most (3 or 4) hostility items were answered affirmative. | This score was computed after multiple imputation of scores indicating the occurrence of abuse and severe maladaptive parenting.  The abuse score was rated missing when no questions concerning abuse were answered.  The severe maladaptive parenting score was rated missing when no questions concerning severe harsh parenting were answered.  . |

* = variable with a high amount of missing values, i.e. only included in subset A.

**References**

Crown, S., & Crisp, A. H. (1979). *Manual of the Crown-Crisp Experiential Index*. London: Hodder & Stroughton.

Dunn, J., Deater-Deckard, K., Pickering, K., & Golding, J. (1999). Siblings, parents, and partners: Family relationships within a longitudinal community study. *Journal of Child Psychology and Psychiatry*, *40*(7), 1025-1038.

Fullard, W., McDevitt, S., & Carey, W. (1984) Assessing temperament in one to three year old children. *Journal of Pediatric Psychology*, *9*, 205–216.

Lereya, S. T., Copeland, W. E., Costello, E. J., & Wolke, D. (2015). Adult mental health consequences of peer bullying and maltreatment in childhood: Two cohorts in two countries. *The Lancet Psychiatry*, *2*(6), 524–531.

Wechsler, D., Golombok, S., & Rust, J. (1992). *WISC-III UK Wechsler Intelligence Scale for Children – Third Edition UK Manual*. Sidcup, UK: The Psychological Corporation.

Wilhelm, K., & Parker, G. (1988). The Development of a Measure of Intimate Bonds. *Psychological Medicine*, *18*(1), 225-234.

Breider, S., Hoekstra, P. J., Wardenaar, K., Van den Hoofdakker, B. J., Dietrich, A., & De Bildt, A. Early-life environmental and child factors associated with the presence of disruptive behaviors in seven-year-old children with autistic traits in the Avon Longitudinal Study of Parents and Children. J Autism Dev Disord. S. Breider at Department of Child and Adolescent Psychiatry, University Medical Center Groningen, University of Groningen, Groningen, The Netherlands, s.breider@accare.nl.
