# Supplementary material for: Early-Life Environmental and Child Factors Associated with the Presence of Disruptive Behaviors in Seven-Year-Old Children with Autistic Traits in the Avon Longitudinal Study of Parents and Children
Source: J Autism Dev Disord. 2021 Jul 10;52(6):2747–61. doi: 10.1007/s10803-021-05081-x (PMC9114014; doi:10.1007/s10803-021-05081-x)
Supplement: Supplementary file 2 — Supplementary file2 (DOCX 18 kb) [file 10803_2021_5081_MOESM2_ESM.docx]

**Online Resource Table 2** Comparison of level of autistic traits and disruptive behavior between participants in subset A and participants excluded from subset A due to a high amount of missing values on independent variables, for the ASD+DB_,_ ASD–DB, and control group

|  | ASD+DB | | | | | | | |  | ASD–DB | | | | | | | |  | Controls | | | | | | | |
| --- | --- | --- | --- | --- | --- | --- | --- | --- | --- | --- | --- | --- | --- | --- | --- | --- | --- | --- | --- | --- | --- | --- | --- | --- | --- | --- |
|  | Subset A  (*n* = 178) | |  | Excluded from subset A  (*n* = 129) | |  |  |  |  | Subset A  (*n* = 120) | |  | Excluded from subset A  (*n* = 78) | |  |  |  |  | Subset A  (*n* = 3,385) | |  | Excluded from subset A  (*n* = 2,511) | |  |  |  |
|  | *M* | *SD* |  | *M* | *SD* |  | *t* | *p* |  | *M* | *SD* |  | *M* | *SD* |  | *t* | *p* |  | *M* | *SD* |  | *M* | *SD* |  | *t* | *p* |
| SCDC | 13.0 | 3.69 |  | 14.2 | 4.54 |  | 2.53 | .01 |  | 11.3 | 2.75 |  | 11.3 | 2.93 |  | 0.17 | .86 |  | 1.80 | 2.07 |  | 1.72 | 2.06 |  | -1.43 | .15 |
| SDQ_EXT_ | 11.8 | 2.20 |  | 11.9 | 2.33 |  | 0.44 | .66 |  | 6.00 | 1.85 |  | 6.14 | 2.04 |  | 0.47 | .64 |  | 3.83 | 2.27 |  | 3.90 | 2.29 |  | 1.19 | .24 |

ASD = Autism Spectrum Disorder; DB = disruptive behavior; Controls = no autistic traits and no disruptive behaviors; SCDC = Social Communication Disorder Checklist (i.e. autistic traits); SDQ_EXT_ = sum of the Conduct and Hyperactivity subscale of the Strengths and Difficulties Questionnaire (i.e. disruptive behaviors).

Breider, S., Hoekstra, P. J., Wardenaar, K., Van den Hoofdakker, B. J., Dietrich, A., & De Bildt, A. Early-life environmental and child factors associated with the presence of disruptive behaviors in seven-year-old children with autistic traits in the Avon Longitudinal Study of Parents and Children. J Autism Dev Disord. S. Breider at Department of Child and Adolescent Psychiatry, University Medical Center Groningen, University of Groningen, Groningen, The Netherlands, s.breider@accare.nl.
