# Supplementary material for: Early-Life Environmental and Child Factors Associated with the Presence of Disruptive Behaviors in Seven-Year-Old Children with Autistic Traits in the Avon Longitudinal Study of Parents and Children
Source: J Autism Dev Disord. 2021 Jul 10;52(6):2747–61. doi: 10.1007/s10803-021-05081-x (PMC9114014; doi:10.1007/s10803-021-05081-x)
Supplement: Supplementary file 3 — Supplementary file3 (DOCX 18 kb) [file 10803_2021_5081_MOESM3_ESM.docx]

**Online Resource Table 3** Comparison of demographic characteristics between participants in subset A and participants excluded

from subset A due to a high amount of missing values on independent variables

|  | Subset A  (*n* = 3,683) | |  | Excluded from subset A  (*n* = 2,718) | |  |  |  |
| --- | --- | --- | --- | --- | --- | --- | --- | --- |
|  |  | *n* |  |  | *n* |  | Test-statistic | *p* |
| Sex: % male | 49.5 | 3,683 |  | 50.7 | 2,718 |  | χ2 = 1.00 | .32 |
| IQ: mean (*SD*) | 108 (15.7) | 3,053 |  | 105 (15.1) | 1,818 |  | *t* = -5.68 | < .001 |
| Social class mothers during pregnancy: |  | 3,254 |  |  | 1,944 |  | χ2 = 27.9 | < .001 |
| % High | 43.4 |  |  | 36.0 |  |  |  |  |
| % Medium | 48.6 |  |  | 54.5 |  |  |  |  |
| % Low | 7.99 |  |  | 9.52 |  |  |  |  |
| Social class partners during pregnancy: |  | 3,377 |  |  | 2,098 |  | χ2 = 24.1 | < .001 |
| % High | 47.1 |  |  | 40.5 |  |  |  |  |
| % Medium | 45.7 |  |  | 50.8 |  |  |  |  |
| % Low | 7.17 |  |  | 8.72 |  |  |  |  |
| Family: % two-parent household in the child’s first year | 98.8 | 3,662 |  | 90.4 | 2,492 |  | χ2 = 239 | < .001 |
| Ethnicity: % white | 97.5 | 3,661 |  | 95.2 | 2,538 |  | χ2 = 23.6 | < .001 |

Breider, S., Hoekstra, P. J., Wardenaar, K., Van den Hoofdakker, B. J., Dietrich, A., & De Bildt, A. Early-life environmental and child factors associated with the presence of disruptive behaviors in seven-year-old children with autistic traits in the Avon Longitudinal Study of Parents and Children. J Autism Dev Disord. S. Breider at Department of Child and Adolescent Psychiatry, University Medical Center Groningen, University of Groningen, Groningen, The Netherlands, s.breider@accare.nl.
