# Supplementary material for: Early-Life Environmental and Child Factors Associated with the Presence of Disruptive Behaviors in Seven-Year-Old Children with Autistic Traits in the Avon Longitudinal Study of Parents and Children
Source: J Autism Dev Disord. 2021 Jul 10;52(6):2747–61. doi: 10.1007/s10803-021-05081-x (PMC9114014; doi:10.1007/s10803-021-05081-x)
Supplement: Supplementary file 4 — Supplementary file4 (DOCX 18 kb) [file 10803_2021_5081_MOESM4_ESM.docx]

**Online Resource Table 4** Comparison of demographic characteristics between participants in subset B and ALSPAC participants

excluded from the current study due to missing data on autistic traits and/or disruptive behaviors

|  | Subset B  (*n* = 6,401) | |  | ALSPAC participants excluded due to  missing data on autistic traits and/or disruptive behaviors (*n* = 8,031) | |  |  |  |
| --- | --- | --- | --- | --- | --- | --- | --- | --- |
|  |  | *n* |  |  | *n* |  | Test-statistic | *p* |
| Sex: % male | 50.0 | 6,401 |  | 51.3 | 7,450 |  | χ2 = 2.45 | .12 |
| IQ: mean (*SD*) | 107 (15.5) | 4,871 |  | 101 (15.5) | 1,771 |  | *t* = -14.0 | < .001 |
| Social class mothers during pregnancy: |  | 5,198 |  |  | 3,729 |  | χ2 = 226 | < .001 |
| % High | 40.6 |  |  | 28.4 |  |  |  |  |
| % Medium | 50.8 |  |  | 54.5 |  |  |  |  |
| % Low | 8.56 |  |  | 17.0 |  |  |  |  |
| Social class partners during pregnancy: |  | 5,475 |  |  | 4,145 |  | χ2 = 136 | < .001 |
| % High | 44.6 |  |  | 33.9 |  |  |  |  |
| % Medium | 47.7 |  |  | 53.8 |  |  |  |  |
| % Low | 7.76 |  |  | 12.3 |  |  |  |  |
| Family % two-parent household in the child’s first year | 95.4 | 6,154 |  | 89.0 | 4,212 |  | χ2 = 156 | < .001 |
| Ethnicity: % white | 96.6 | 6,199 |  | 93.1 | 5,044 |  | χ2 = 71.8 | < .001 |

Breider, S., Hoekstra, P. J., Wardenaar, K., Van den Hoofdakker, B. J., Dietrich, A., & De Bildt, A. Early-life environmental and child factors associated with the presence of disruptive behaviors in seven-year-old children with autistic traits in the Avon Longitudinal Study of Parents and Children. J Autism Dev Disord. S. Breider at Department of Child and Adolescent Psychiatry, University Medical Center Groningen, University of Groningen, Groningen, The Netherlands, s.breider@accare.nl.
