# Supplementary material for: Early-Life Environmental and Child Factors Associated with the Presence of Disruptive Behaviors in Seven-Year-Old Children with Autistic Traits in the Avon Longitudinal Study of Parents and Children
Source: J Autism Dev Disord. 2021 Jul 10;52(6):2747–61. doi: 10.1007/s10803-021-05081-x (PMC9114014; doi:10.1007/s10803-021-05081-x)
Supplement: Supplementary file 5 — Supplementary file5 (DOCX 76 kb) [file 10803_2021_5081_MOESM5_ESM.docx]

**Online Resource Table 5** Lasso logistic regression results per group comparison in subset A and B across imputation sets: range of explained deviance of the model, range of used lambdas, and range and number of non-zero *b*-coefficients per independent variable

|  | ASD+DB versus ASD–DB | | | | | | |  | ASD+DB versus controls | | | | | | |  | ASD–DB versus controls | | | | | | |
| --- | --- | --- | --- | --- | --- | --- | --- | --- | --- | --- | --- | --- | --- | --- | --- | --- | --- | --- | --- | --- | --- | --- | --- |
|  | Subset A | | |  | Subset B | | |  | Subset A | | |  | Subset B | | |  | Subset A | | |  | Subset B | | |
| Range of explained deviance of the total model (in percentages, min – max) | 22.5 | 30.6 |  |  | 16.0 | 19.6 |  |  | 33.1 | 35.1 |  |  | 29.2 | 31.2 |  |  | 7.61 | 12.4 |  |  | 6.58 | 8.74 |  |
| Range of lambdas (min – max) | 0.0236 | 0.0318 |  |  | 0.0200 | 0.0237 |  |  | 0.0032 | 0.0042 |  |  | 0.0020 | 0.0029 |  |  | 0.0037 | 0.0055 |  |  | 0.0026 | 0.0038 |  |
|  | *b* min | *b* max | nb |  | *b* min | *b* max | nb |  | *b* min | *b* max | nb |  | *b* min | *b* max | nb |  | *b* min | *b* max | nb |  | *b* min | *b* max | nb |
| *Family demographics* | | | | | | | | | | | | | | | | | | | | | | | |
| Maternal age at time of birth (reference = middle [20-29]) |  |  |  |  |  |  |  |  |  |  |  |  |  |  |  |  |  |  |  |  |  |  |  |
| - Low (< 20) |  |  |  |  |  |  |  |  |  |  |  |  | 0.0151 | 0.5152 | 9 |  |  |  |  |  |  |  |  |
| - High (> 29) |  |  |  |  |  |  |  |  |  |  |  |  |  |  |  |  |  |  |  |  |  |  |  |
| High partner’s age at time of birth (> 35) | -0.0039 | -0.2490 | 9 |  |  |  |  |  |  |  |  |  |  |  |  |  |  |  |  |  |  |  |  |
| Firstborn child | 0.0180 | 0.1662 | 8 |  |  |  |  |  |  |  |  |  |  |  |  |  |  |  |  |  |  |  |  |
| Summer birth | -0.1789 | -0.2819 | 10 |  | -0.2311 | -0.3346 | 10 |  | -0.0193 | -0.1079 | 10 |  |  |  |  |  |  |  |  |  | 0.0058 | 0.0807 | 9 |
| Twin birth |  |  |  |  |  |  |  |  | -0.2972 | -0.7288 | 10 |  | -0.3564 | -0.7472 | 10 |  |  |  |  |  |  |  |  |
| Maternal education (reference = CSE/none) |  |  |  |  |  |  |  |  |  |  |  |  |  |  |  |  |  |  |  |  |  |  |  |
| - Vocational |  |  |  |  |  |  |  |  |  |  |  |  |  |  |  |  |  |  |  |  |  |  |  |
| - O level |  |  |  |  |  |  |  |  |  |  |  |  |  |  |  |  |  |  |  |  |  |  |  |
| - A level |  |  |  |  |  |  |  |  |  |  |  |  | -0.0172 | -0.0777 | 7 |  | 0.0384 | 0.0914 | 9 |  |  |  |  |
| - Degree |  |  |  |  |  |  |  |  |  |  |  |  |  |  |  |  |  |  |  |  |  |  |  |
| Partner’s education (reference = CSE/none) |  |  |  |  |  |  |  |  |  |  |  |  |  |  |  |  |  |  |  |  |  |  |  |
| - Vocational | 0.0073 | 0.2914 | 9 |  | 0.1039 | 0.4218 | 10 |  |  |  |  |  |  |  |  |  |  |  |  |  | -0.0473 | -0.2879 | 10 |
| - O level |  |  |  |  | 0.0134 | 0.1629 | 8 |  |  |  |  |  | 0.0844 | 0.1995 | 10 |  |  |  |  |  |  |  |  |
| - A level |  |  |  |  |  |  |  |  |  |  |  |  |  |  |  |  |  |  |  |  |  |  |  |
| - Degree |  |  |  |  |  |  |  |  |  |  |  |  |  |  |  |  |  |  |  |  |  |  |  |
| Maternal social class (reference = high) |  |  |  |  |  |  |  |  |  |  |  |  |  |  |  |  |  |  |  |  |  |  |  |
| - Medium |  |  |  |  |  |  |  |  |  |  |  |  |  |  |  |  |  |  |  |  |  |  |  |
| - Low |  |  |  |  |  |  |  |  | 0.0539 | 0.5330 | 10 |  | 0.0421 | 0.1875 | 7 |  |  |  |  |  |  |  |  |
| Partner’s social class (reference = high) |  |  |  |  |  |  |  |  |  |  |  |  |  |  |  |  |  |  |  |  |  |  |  |
| - Medium | 0.0007 | 0.2187 | 6 |  | 0.1171 | 0.3444 | 10 |  | 0.0239 | 0.2091 | 10 |  |  |  |  |  |  |  |  |  |  |  |  |
| - Low |  |  |  |  |  |  |  |  |  |  |  |  | -0.0098 | -0.2816 | 8 |  |  |  |  |  |  |  |  |
| Single household at 8 months ^z^ | 0.0387 | 1.1223 | 10 |  |  |  |  |  |  |  |  |  | -0.0289 | -0.2890 | 7 |  | -0.0004 | -0.1105 | 6 |  |  |  |  |
| Single household at 21 months |  |  |  |  |  |  |  |  |  |  |  |  |  |  |  |  |  |  |  |  |  |  |  |
| Single household at 33 months |  |  |  |  |  |  |  |  |  |  |  |  |  |  |  |  |  |  |  |  |  |  |  |
| Single household at 47 months |  |  |  |  |  |  |  |  | 0.0280 | 0.3257 | 10 |  |  |  |  |  |  |  |  |  | 0.0095 | 0.2635 | 10 |
| Number of child’s siblings 18 months ^c^ |  |  |  |  | 0.0108 | 0.0649 | 8 |  | 0.0296 | 0.1482 | 10 |  | 0.0015 | 0.1385 | 10 |  |  |  |  |  |  |  |  |
| Number of child’s siblings 30 months ^c^ |  |  |  |  |  |  |  |  |  |  |  |  |  |  |  |  |  |  |  |  |  |  |  |
|  | | | | | | | | | | | | | | | | | | | | | | | |
| *Pregnancy* | | | | | | | | | | | | | | | | | | | | | | | |
| Infections in first trimester |  |  |  |  | -0.0224 | -0.2124 | 10 |  |  |  |  |  |  |  |  |  |  |  |  |  |  |  |  |
| Infections in second trimester |  |  |  |  | 0.0036 | 0.1474 | 9 |  | 0.0185 | 0.0885 | 10 |  | 0.0211 | 0.1094 | 8 |  |  |  |  |  |  |  |  |
| Infections in third trimester |  |  |  |  |  |  |  |  |  |  |  |  |  |  |  |  |  |  |  |  |  |  |  |
| Maternal alcohol use |  |  |  |  |  |  |  |  |  |  |  |  |  |  |  |  | -0.0282 | -0.1301 | 9 |  |  |  |  |
| Smoking mother | 0.0630 | 0.3152 | 10 |  | 0.0129 | 0.0937 | 8 |  | 0.0982 | 0.2148 | 10 |  | 0.0991 | 0.2140 | 10 |  |  |  |  |  |  |  |  |
| Smoking partner | 0.0074 | 0.2693 | 10 |  | 0.0559 | 0.2077 | 10 |  | 0.0823 | 0.2062 | 10 |  | 0.0290 | 0.1713 | 10 |  |  |  |  |  |  |  |  |
| Maternal medication use |  |  |  |  |  |  |  |  |  |  |  |  |  |  |  |  |  |  |  |  |  |  |  |
| Maternal antidepressant use ^z^ | 0.2481 | 1.1763 | 9 |  | 0.1576 | 0.7630 | 6 |  | 0.1787 | 0.7893 | 10 |  |  |  |  |  |  |  |  |  |  |  |  |
| Diabetes | -0.4202 | -0.6959 | 10 |  | -0.0964 | -0.2925 | 8 |  |  |  |  |  |  |  |  |  | 0.1113 | 0.3651 | 10 |  |  |  |  |
| Maternal pre-pregnancy weight (kg) ^c^ |  |  |  |  |  |  |  |  |  |  |  |  |  |  |  |  |  |  |  |  |  |  |  |
| High level of street traffic |  |  |  |  | 0.0057 | 0.1332 | 9 |  | 0.0549 | 0.1273 | 10 |  | 0.0389 | 0.1982 | 10 |  |  |  |  |  |  |  |  |
| Weekly seafood consumption | -0.2138 | -0.3103 | 10 |  | -0.0293 | -0.1954 | 10 |  | -0.1216 | -0.2124 | 10 |  | -0.2895 | -0.4127 | 10 |  |  |  |  |  |  |  |  |
| Folic acid intake |  |  |  |  | 0.0587 | 0.3284 | 10 |  |  |  |  |  | 0.0062 | 0.1882 | 10 |  |  |  |  |  |  |  |  |
| Maternal prenatal anxiety ^c^ |  |  |  |  |  |  |  |  |  |  |  |  |  |  |  |  |  |  |  |  |  |  |  |
| Maternal prenatal depression ^c^ |  |  |  |  |  |  |  |  |  |  |  |  |  |  |  |  |  |  |  |  |  |  |  |
| Prenatal stress (life events) ^c^ |  |  |  |  | 0.0029 | 0.0092 | 10 |  | 0.0147 | 0.0240 | 10 |  | 0.0091 | 0.0165 | 10 |  |  |  |  |  |  |  |  |
| Maternal external locus of control ^c^ |  |  |  |  |  |  |  |  |  |  |  |  | 0.0047 | 0.0352 | 10 |  |  |  |  |  |  |  |  |
| Affection between parents ^c^ |  |  |  |  |  |  |  |  |  |  |  |  |  |  |  |  |  |  |  |  | -0.0099 | -0.0224 | 10 |
| Aggression between parents  ^c^ | 0.0482 | 0.1056 | 10 |  |  |  |  |  |  |  |  |  |  |  |  |  |  |  |  |  |  |  |  |
| Maternal non-positive pregnancy feelings |  |  |  |  |  |  |  |  |  |  |  |  | -0.0102 | -0.0448 | 6 |  |  |  |  |  |  |  |  |
| Pre-eclampsia | -0.0504 | -0.3247 | 9 |  |  |  |  |  |  |  |  |  |  |  |  |  | 0.0339 | 0.2127 | 7 |  |  |  |  |
| Vaginal bleeding in pregnancy |  |  |  |  |  |  |  |  | -0.1656 | -0.2593 | 10 |  | -0.0437 | -0.1711 | 10 |  |  |  |  |  |  |  |  |
| Premature birth (<37 weeks) |  |  |  |  |  |  |  |  | 0.0016 | 0.1587 | 8 |  | 0.0013 | 0.2129 | 10 |  |  |  |  |  |  |  |  |
| Low birthweight (<2500 grams) | 0.6628 | 0.9505 | 10 |  |  |  |  |  |  |  |  |  |  |  |  |  | -0.1547 | -0.3106 | 9 |  |  |  |  |
|  | | | | | | | | | | | | | | | | | | | | | | | |
| *Delivery* | | | | | | | | | | | | | | | | | | | | | | | |
| Breech presentation before labor |  |  |  |  | - | - | - |  |  |  |  |  | - | - | - |  | -0.0031 | -0.2025 | 6 |  | - | - | - |
| Breech presentation at onset of labor |  |  |  |  | - | - | - |  |  |  |  |  | - | - | - |  |  |  |  |  | - | - | - |
| Breech presentation at delivery/ cesarean section | 0.1098 | 1.0457 | 6 |  | - | - | - |  |  |  |  |  | - | - | - |  |  |  |  |  | - | - | - |
| Cesarean section |  |  |  |  | - | - | - |  |  |  |  |  | - | - | - |  |  |  |  |  | - | - | - |
| Breech birth ^z^ |  |  |  |  | - | - | - |  |  |  |  |  | - | - | - |  |  |  |  |  | - | - | - |
| Maternal hemorrhage prior to delivery |  |  |  |  | - | - | - |  |  |  |  |  | - | - | - |  |  |  |  |  | - | - | - |
| Precipitate labor |  |  |  |  | - | - | - |  |  |  |  |  | - | - | - |  |  |  |  |  | - | - | - |
| Umbilical cord complications |  |  |  |  | - | - | - |  |  |  |  |  | - | - | - |  |  |  |  |  | - | - | - |
|  |  |  |  |  |  |  |  |  |  |  |  |  |  |  |  |  |  |  |  |  |  |  |  |
| *Neonatal* | | | | | | | | | | | | | | | | | | | | | | | |
| Low Apgar at 5 minutes (<7) |  |  |  |  | - | - | - |  |  |  |  |  | - | - | - |  |  |  |  |  | - | - | - |
| Child’s age at discharge from hospital  ^c^ |  |  |  |  | - | - | - |  |  |  |  |  | - | - | - |  |  |  |  |  | - | - | - |
| Anemia | -0.0510 | -0.2883 | 10 |  | -0.1388 | -0.2263 | 10 |  | -0.0982 | -0.2131 | 10 |  | -0.1575 | -0.2833 | 10 |  |  |  |  |  |  |  |  |
| Feeding problems | 0.0525 | 0.8140 | 10 |  | - | - | - |  |  |  |  |  | - | - | - |  | -0.1019 | -0.2742 | 6 |  | - | - | - |
| Oxygen problems |  |  |  |  | - | - | - |  |  |  |  |  | - | - | - |  |  |  |  |  | - | - | - |
| Breastfeeding in first four weeks |  |  |  |  |  |  |  |  |  |  |  |  |  |  |  |  |  |  |  |  |  |  |  |
| Duration of breastfeeding (reference = never) |  |  |  |  |  |  |  |  |  |  |  |  |  |  |  |  |  |  |  |  |  |  |  |
| - < 3 months |  |  |  |  |  |  |  |  |  |  |  |  |  |  |  |  |  |  |  |  |  |  |  |
| - 3- 5 months |  |  |  |  |  |  |  |  |  |  |  |  |  |  |  |  |  |  |  |  |  |  |  |
| - ≥ 6 months |  |  |  |  |  |  |  |  |  |  |  |  |  |  |  |  |  |  |  |  |  |  |  |
|  | | | | | | | | | | | | | | | | | | | | | | | |
| *Child* | | | | | | | | | | | | | | | | | | | | | | | |
| Intelligence ^c^ |  |  |  |  |  |  |  |  | -0.0066 | -0.0144 | 10 |  | -0.0140 | -0.0217 | 10 |  | -0.0032 | -0.0131 | 9 |  | -0.0033 | -0.0143 | 10 |
| Non-white ethnicity |  |  |  |  |  |  |  |  |  |  |  |  | -0.0731 | -0.4424 | 10 |  |  |  |  |  | -0.0271 | -0.1764 | 6 |
| Male sex | 0.2554 | 0.4308 | 10 |  | 0.3835 | 0.4458 | 10 |  | 0.7883 | 0.8888 | 10 |  | 0.8670 | 0.9292 | 10 |  | 0.1137 | 0.2368 | 10 |  | 0.2338 | 0.3128 | 10 |
| Temperament: Activity ^c^ |  |  |  |  | 0.0258 | 0.0443 | 10 |  | 0.0255 | 0.0334 | 10 |  | 0.0310 | 0.0528 | 10 |  |  |  |  |  |  |  |  |
| Temperament: Rhythmicity ^c^ |  |  |  |  |  |  |  |  |  |  |  |  |  |  |  |  |  |  |  |  |  |  |  |
| Temperament: Approach ^c^ | -0.0132 | -0.0192 | 10 |  | -0.0005 | -0.0070 | 10 |  | -0.0057 | -0.0112 | 10 |  | -0.0026 | -0.0060 | 8 |  |  |  |  |  |  |  |  |
| Temperament: Adaptability ^c^ | 0.0493 | 0.0646 | 10 |  | 0.0150 | 0.0360 | 10 |  | 0.0646 | 0.0717 | 10 |  | 0.0416 | 0.0495 | 10 |  |  |  |  |  |  |  |  |
| Temperament: Intensity ^c^ | 0.0456 | 0.0590 | 10 |  | 0.0370 | 0.0483 | 10 |  | 0.0521 | 0.0616 | 10 |  | 0.0484 | 0.0623 | 10 |  |  |  |  |  |  |  |  |
| Temperament: Mood ^c^ |  |  |  |  |  |  |  |  |  |  |  |  |  |  |  |  | 0.0341 | 0.0391 | 10 |  | 0.0045 | 0.0182 | 10 |
| Temperament: Persistence ^c^ | 0.0168 | 0.0263 | 10 |  | 0.0050 | 0.0153 | 10 |  | 0.0261 | 0.0297 | 10 |  | 0.0240 | 0.0365 | 10 |  |  |  |  |  | 0.0008 | 0.0085 | 8 |
| Temperament: Distractibility ^c^ |  |  |  |  |  |  |  |  |  |  |  |  |  |  |  |  |  |  |  |  |  |  |  |
| Temperament: Threshold ^c^ | -0.0342 | -0.0451 | 10 |  | -0.0372 | -0.0529 | 10 |  | -0.0374 | -0.0460 | 10 |  | -0.0475 | -0.0583 | 10 |  |  |  |  |  |  |  |  |
|  | | | | | | | | | | | | | | | | | | | | | | | |
| *Preschool parental and family* | | | | | | | | | | | | | | | | | | | | | | | |
| Mother’s experienced social support ^c^ |  |  |  |  |  |  |  |  |  |  |  |  |  |  |  |  |  |  |  |  |  |  |  |
| Affection between parents 8 months,  mother rated ^c^ |  |  |  |  |  |  |  |  |  |  |  |  |  |  |  |  |  |  |  |  | -0.0004 | -0.0050 | 6 |
| Aggression between parents 8 months,  mother rated ^c^ |  |  |  |  |  |  |  |  | 0.0218 | 0.0510 | 10 |  | 0.0018 | 0.0208 | 6 |  |  |  |  |  |  |  |  |

| Affection between parents 8 months,  partner rated ^c^ |  |  |  |  | - | - | - |  |  |  |  |  | - | - | - |  | -0.0011 | -0.0155 | 10 |  | - | - | - |
| --- | --- | --- | --- | --- | --- | --- | --- | --- | --- | --- | --- | --- | --- | --- | --- | --- | --- | --- | --- | --- | --- | --- | --- |
| Aggression between parents 8 months,  partner rated ^c^ |  |  |  |  | - | - | - |  |  |  |  |  | - | - | - |  |  |  |  |  | - | - | - |
| Warmth between parents 33 months,  mother rated ^c^ | -0.0010 | -0.0077 | 8 |  |  |  |  |  | -0.0207 | -0.0277 | 10 |  | -0.0077 | -0.0231 | 10 |  | -0.0003 | -0.0084 | 8 |  | -0.0019 | -0.0110 | 10 |
| Rows between parents 33 months,  mother rated ^c^ |  |  |  |  |  |  |  |  |  |  |  |  |  |  |  |  |  |  |  |  |  |  |  |
| Warmth between parents 33 months,  partner rated ^c^ |  |  |  |  | - | - | - |  |  |  |  |  | - | - | - |  |  |  |  |  | - | - | - |
| Rows between parents 33 months,  partner rated ^c^ |  |  |  |  | - | - | - |  |  |  |  |  | - | - | - |  | 0.0279 | 0.0547 | 10 |  | - | - | - |
| Maternal anxiety ^c^ |  |  |  |  |  |  |  |  |  |  |  |  |  |  |  |  |  |  |  |  |  |  |  |
| Partner’s anxiety ^c^ |  |  |  |  |  |  |  |  |  |  |  |  |  |  |  |  | 0.0104 | 0.0306 | 10 |  | 0.0114 | 0.0492 | 10 |
| Maternal depression ^c^ |  |  |  |  |  |  |  |  | 0.0029 | 0.0169 | 8 |  | 0.0089 | 0.0225 | 10 |  | 0.0125 | 0.0389 | 10 |  | 0.0182 | 0.0305 | 10 |
| Partner’s depression ^c^ |  |  |  |  |  |  |  |  |  |  |  |  | 0.0125 | 0.0467 | 10 |  |  |  |  |  |  |  |  |
| Stress (life events) ^c^ |  |  |  |  |  |  |  |  |  |  |  |  |  |  |  |  | 0.0018 | 0.0068 | 10 |  | 0.0077 | 0.0133 | 10 |
| Maternal antisocial behavior ^c^ | 0.0014 | 0.0786 | 10 |  | 0.0221 | 0.0944 | 10 |  | 0.1076 | 0.1446 | 10 |  | 0.0866 | 0.1361 | 10 |  |  |  |  |  |  |  |  |
| Accident prevention measures ^c^ | -0.0492 | -0.0895 | 10 |  | -0.0053 | -0.0662 | 10 |  |  |  |  |  |  |  |  |  | 0.0353 | 0.0709 | 10 |  | 0.0125 | 0.0244 | 8 |
| Parenting score 6 months mother ^c^ | -0.1247 | -0.1827 | 10 |  | -0.0297 | -0.0651 | 10 |  | -0.0074 | -0.0377 | 10 |  |  |  |  |  |  |  |  |  |  |  |  |
| Parenting score 6 months partner ^c^ |  |  |  |  | -0.0244 | -0.0362 | 10 |  |  |  |  |  |  |  |  |  |  |  |  |  |  |  |  |
| Parenting score 18 months mother ^c^ |  |  |  |  |  |  |  |  |  |  |  |  |  |  |  |  |  |  |  |  |  |  |  |
| Parenting score 18 months partner ^c^ |  |  |  |  |  |  |  |  | -0.0096 | -0.0166 | 10 |  | -0.0029 | -0.0108 | 9 |  |  |  |  |  |  |  |  |
| Parenting score 38 months mother ^c^ |  |  |  |  |  |  |  |  |  |  |  |  | 0.0237 | 0.0404 | 10 |  |  |  |  |  |  |  |  |
| Parenting score 38 months partner ^c^ | -0.0215 | -0.0404 | 10 |  |  |  |  |  |  |  |  |  | -0.0042 | -0.0146 | 9 |  |  |  |  |  |  |  |  |
| Parental bonding 8 months mother  ^c^ |  |  |  |  |  |  |  |  |  |  |  |  |  |  |  |  |  |  |  |  |  |  |  |
| Parental bonding 8 months partner  ^c^ |  |  |  |  | - | - | - |  | 0.0067 | 0.0382 | 10 |  | - | - | - |  |  |  |  |  | - | - | - |
| Parental bonding 33 months mother ^c^ | -0.0125 | -0.0308 | 10 |  | -0.0209 | -0.0348 | 10 |  | -0.0443 | -0.0530 | 10 |  | -0.0673 | -0.0788 | 10 |  | -0.0087 | -0.0239 | 10 |  | -0.0101 | -0.0265 | 10 |
| Positive parenting experience score 21 months mother ^c^ |  |  |  |  | -0.0004 | -0.0183 | 8 |  |  |  |  |  |  |  |  |  |  |  |  |  |  |  |  |
| Negative parenting experience score 21 months mother ^c^ |  |  |  |  |  |  |  |  |  |  |  |  |  |  |  |  |  |  |  |  |  |  |  |
| Positive parenting experience score 21 months partner ^c^ |  |  |  |  | - | - | - |  |  |  |  |  | - | - | - |  |  |  |  |  | - | - | - |
| Negative parenting experience score 21 months partner ^c^ |  |  |  |  | - | - | - |  |  |  |  |  | - | - | - |  |  |  |  |  | - | - | - |
| Positivity scale score 47 months mother ^c^ | -0.0741 | -0.1119 | 10 |  | -0.0973 | -0.1278 | 10 |  | -0.1148 | -0.1540 | 10 |  | -0.1704 | -0.2046 | 10 |  |  |  |  |  |  |  |  |
| Negativity scale score 47 months mother ^c^ | 0.0333 | 0.0655 | 10 |  | 0.0337 | 0.0617 | 10 |  | 0.2165 | 0.2352 | 10 |  | 0.2071 | 0.2412 | 10 |  | 0.1371 | 0.1600 | 10 |  | 0.1508 | 0.1757 | 10 |
| Positivity scale score 47 months partner ^c^ | -0.1114 | -0.2717 | 10 |  | - | - | - |  | -0.0059 | -0.1150 | 10 |  | - | - | - |  | 0.0446 | 0.1944 | 7 |  | - | - | - |
| Negativity scale score 47 months partner ^c^ | 0.0012 | 0.0697 | 10 |  | - | - | - |  | 0.1282 | 0.1847 | 10 |  | - | - | - |  | 0.0305 | 0.0907 | 10 |  | - | - | - |
| Child maltreatment | 0.0112 | 0.1278 | 8 |  |  |  |  |  | 0.2192 | 0.2597 | 10 |  | 0.1584 | 0.2062 | 10 |  | 0.0629 | 0.1013 | 10 |  | 0.1589 | 0.1872 | 10 |

ASD = Autism Spectrum Disorder; DB = disruptive behavior; controls = no ASD and no DB. Subset A: i.e., participants with a high amount of missing values excluded; *n* = 3,683, *n* ASD+DB = 178, *n* ASD–DB = 120, *n* controls = 3,385; 100 independent variables. Subset B: i.e., variables with a high amount of missing values excluded; *n* = 6,401, *n* ASD+DB = 307, *n* ASD–DB = 198, *n* controls = 5,896; 79 independent variables. Variables which were not included in subset B are indicated with a ‘-’. Range of lambdas = range of used lambdas across the ten imputation sets; for each imputation set, the lambda with the smallest mean cross-validated error over 1000 iterations was used. nb = number of imputation sets in which a variable has a non-zero *b*-coefficient. ^c^ = continuous variable. ^z^ = a variable category was empty in a number of imputation sets; this always concerned ASD–DB in subset A. *b*-coefficients are only displayed for variables with ≥ six non-zero *b*-coefficients over the ten imputation sets. Shaded coefficients (in grey) indicate that a variable is assumed to be associated with group membership for the concerning group comparison, i.e. had ≥ six non-zero *b*-coefficients in subset A and B, or, for variables only included in subset A, had ≥ six non-zero *b*-coefficients in subset A. For each group comparison, the reference group is the second named group: a positive *b*-coefficient indicates higher odds of being in the first named group when a variable was present (for a categorical variable) or had a higher score (in case of a continuous variable). A *b*-coefficient indicates the increase of log-odds when an independent variable increases with one.

Breider, S., Hoekstra, P. J., Wardenaar, K., Van den Hoofdakker, B. J., Dietrich, A., & De Bildt, A. Early-life environmental and child factors associated with the presence of disruptive behaviors in seven-year-old children with autistic traits in the Avon Longitudinal Study of Parents and Children. J Autism Dev Disord. S. Breider at Department of Child and Adolescent Psychiatry, University Medical Center Groningen, University of Groningen, Groningen, The Netherlands, s.breider@accare.nl.
